# An Avalanche Effect Nanomodulator Triggers Cascade Ferroptosis/Cuproptosis for Immunosuppressive Microenvironment Remodeling

**DOI:** 10.1002/advs.77120

**Published:** 2026-08-13

**Authors:** Hongmei Zhou, Caixi Yu, Guilan Xu, Lijuan Liu, Yujia Liang, Yulin Liang, He Ding, Litu Zhang, Piaoping Yang, Chen Wang

**Affiliations:** ^1^ Department of Experimental Research University Engineering Research Center of Oncolytic & Nanosystem Development (Guangxi) Guangxi Medical University Cancer Hospital Nanning People's Republic of China; ^2^ Guangxi Key Laboratory of Extremely Weak Magnetic Field in Cancer Medicine Basic and Translational Research for Colorectal Cancer Nanning People's Republic of China; ^3^ Key Laboratory of Superlight Materials and Surface Technology Ministry of Education College of Material Sciences and Chemical Engineering Harbin Engineering University Harbin People's Republic of China; ^4^ Yangtze Delta Region Advanced Research Institute of Harbin Engineering University Harbin Engineering University NanTong People's Republic of China; ^5^ Sanya Nanhai Innovation and Development Base of Harbin Engineering University Sanya People's Republic of China

**Keywords:** alleviating tumor hypoxia, cuproptosis, ferroptosis, immunogenic cell death, immunosuppressive microenvironment, nanomodulator

## Abstract

The tumor immunosuppressive microenvironment severely impedes the efficacy of immunotherapy. An avalanche effect nanomodulator, L‐Arginine/lificiguat@Copper‐hollow Prussian blue@Calcium phosphate nanoparticles, activates immunotherapy and remodels the immunosuppressive microenvironment through cascade‐triggered ferroptosis and cuproptosis. Within the tumor microacidic environment, calcium ions released by the nanomodulator induce mitochondrial calcium overload and membrane potential collapse. Concurrently, the nanomodulator alleviates tumor hypoxia through synergistic nitric oxide production and hypoxia‐inducible factor‐1α signaling pathway inhibition. Under 808 nm laser irradiation, the photothermal effect of the nanomodulator further promotes nitric oxide and iron/copper ions release. Iron and copper ions generate abundant reactive oxygen species through multiple enzyme activities, a process that synergizes with nitric oxide to form a reactive oxygen species/reactive nitrogen species storm, which intensifies lipid peroxidation and glutathione depletion, ultimately inducing ferroptosis and cuproptosis. The two immunogenic cell death pathways of ferroptosis and cuproptosis significantly promote dendritic cell maturation and cytotoxic T lymphocyte infiltration, transforming cold tumors into hot tumors. The nanomodulator effectively remodels the immunosuppressive microenvironment to establish robust systemic antitumor immune memory, thereby inhibiting primary tumor growth and distant metastasis, offering a novel strategy for tumor immunotherapy.

## Graphical abstract

A nanomodulator called L‐Arginine/lificiguat@Copper‐hollow Prussian blue@Calcium phosphate nanoparticles is constructed to launch a self‐amplifying “avalanche effect” in tumor cells. The ensuing cascade, triggered by the acidic tumor microenvironment and near‐infrared light, induces calcium overload, generates a reactive oxygen species and reactive nitrogen species storm, and synchronously initiates ferroptosis and cuproptosis, thereby activating antitumor immunotherapy and remodeling the immunosuppressive microenvironment.

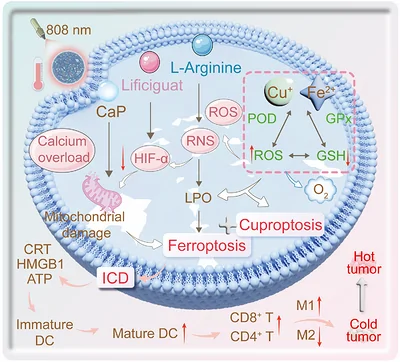

## 1 Introduction

Immunogenic cell death (ICD) activates the host adaptive immune response by releasing damage‐associated molecular patterns (DAMPs), enabling the recognition and attack of tumor cells expressing homologous antigens [1, 2]. However, the clinical efficacy of ICD is often severely limited by the immunosuppressive microenvironment, which is jointly constituted by multiple immunosuppressive cell subsets, cytokine networks, and physicochemical barriers [3, 4, 5, 6]. For instance, the substantial infiltration of myeloid‐derived suppressor cells and regulatory T cells, coupled with the excessive secretion of immunosuppressive factors such as transforming growth factor‐β and interleukin‐10, collectively impede the activation and infiltration of effector T cells [7, 8]. Furthermore, hypoxia within the tumor region activates the hypoxia‐inducible factor‐1α (HIF‐1α) signaling pathway, which not only promotes tumor cell survival and proliferation but also upregulates immune checkpoint molecules like programmed death‐ligand 1, further exacerbating immune escape [9, 10, 11]. Concurrently, the acidic conditions and nutrient depletion within the tumor microenvironment (TME) form physicochemical barriers that significantly restrict the infiltration and function of effector immune cells, ultimately leading to low response rates to immunotherapy [12, 13]. These multidimensional immunosuppressive mechanisms collectively induce a “cold” immune phenotype locally within tumors, characterized by impaired dendritic cell (DCs) maturation, insufficient cytotoxic T lymphocyte infiltration, and compromised immune effector functions. Therefore, effectively remodeling the tumor immunosuppressive microenvironment to transition it from an immunosuppressive to an immune‐supportive state has become a critical scientific challenge in the field of tumor immunotherapy [14, 15].

Ferroptosis and cuproptosis, as two novel forms of programmed cell death, have garnered significant attention due to their unique metabolic characteristics and substantial immune activation potential [16, 17]. Ferroptosis is an iron‐dependent form of cell death characterized by the accumulation of lipid peroxidation (LPO) resulting from the loss of glutathione peroxidase 4 (GPX4) activity, which subsequently triggers oxidative damage to the cell membrane system [18, 19, 20]. Cuproptosis, triggered by excessive copper ions, manifests as direct binding of copper ions to acylated proteins in the tricarboxylic acid cycle, inducing mitochondrial protein toxicity stress and respiratory chain dysfunction [21, 22]. Despite differences in molecular mechanisms and subcellular localization, the antitumor effects of ferroptosis and cuproptosis are closely associated with oxidative stress, disruption of metabolic homeostasis, and mitochondrial damage [23, 24, 25]. Notably, Fe^3+^ catalyzes the conversion of endogenous H_2_O_2_ within tumor cells into hydroxyl radicals, depleting glutathione (GSH) and downregulating GPX4 expression [26, 27]. However, Fe^3+^‐mediated Fenton reactions exhibit higher efficiency under strongly acidic conditions, while their reactivity is limited in the physiologically neutral or weakly acidic TME. Cu^2+^, by comparison, retains high activity under neutral and weakly acidic milieus, efficiently triggering ferroptosis through a Fenton‐like reaction at a rate nearly 160 times faster than that of Fe^3+^ [28]. Additionally, Cu^2+^ can trigger cuproptosis by disrupting mitochondrial thiooxidation metabolism, leading to aggregation of dihydrolipoamide S‐acetyltransferase (DLAT) and depletion of iron‐sulfur (Fe‐S) clusters [29, 30]. Accumulating evidence suggests that the interplay between oxidative stress and nitrosative stress may serve as a critical node for synergistically activating ferroptosis and cuproptosis. The depletion of GSH simultaneously sensitizes tumor cells to iron‐mediated LPO and copper‐induced mitochondrial dysfunction, thereby establishing a positive feedback loop that amplifies organelle damage and ICD [31, 32, 33, 34]. It is worth noting that the combined activation of ferroptosis and cuproptosis has been shown to induce tumor cell death more effectively than targeting either pathway individually [35, 36]. Ferroptosis and cuproptosis further activate ICD, triggering robust immune responses. Cells release DAMPs to activate DCs and promote antigen presentation, such as cell‐surface‐exposed calreticulin (CRT), high‐mobility group protein B1 (HMGB1), and adenosine triphosphate (ATP) exposed on the cell surface [37, 38]. Given the high metabolic plasticity and defense mechanisms of tumor cells, inducing a single death pathway often yields limited effects. Therefore, developing combined strategies that synergistically trigger ferroptosis and cuproptosis holds promise for enhancing antitumor immune responses by amplifying immunogenic signals.

Nitric oxide (NO), as a key gaseous signaling molecule, disrupts the redox homeostasis of tumor cells through multiple pathways within the tumor [39, 40]. On one hand, NO interacts with reactive oxygen species (ROS) to form highly reactive nitrogen species (RNS), exacerbating oxidative damage [41, 42]. NO also modifies antioxidant proteins like GSH reductase through nitrosylation, causing the collapse of intracellular antioxidant systems and thereby promoting LPO and ferroptosis [43, 44]. On the other hand, NO induces mitochondrial dysfunction and endoplasmic reticulum stress, disrupting intracellular calcium homeostasis [45, 46]. Calcium overload further triggers mitochondrial membrane potential collapse and energy metabolism disorders, forming a positive feedback loop that intensifies oxidative damage in tumors [47, 48]. Furthermore, disrupted calcium homeostasis promotes opening of the mitochondrial permeability transition pore, which in turn activates calcium‐dependent proteases and intensifies oxidative stress, thereby leading to cellular structural collapse and the substantial release of DAMPs [49, 50]. Crucially, NO also alleviates tumor hypoxia by inhibiting the HIF‐1α pathway, disrupting hypoxia‐driven expression of immunosuppressive factors, and M2 macrophage polarization [51, 52, 53]. The synergy between calcium overload and NO therapy integrates ionic interference with gaseous signaling regulation, which disrupts tumor cell homeostasis and promotes vascular normalization, thereby fostering a favorable microenvironment for immune cell infiltration.

A nanomodulator with an avalanche effect, called L‐Arginine/lificiguat@Copper‐hollow Prussian blue@Calcium phosphate nanoparticles (LYCHC NPs), has been designed to activate immunotherapy efficiently by triggering ferroptosis and cuproptosis in a cascading manner and remodeling the immunosuppressive microenvironment (Scheme 1). The nanomodulator uses copper‐doped hollow Prussian blue nanoparticles (CHP NPs) as its core carrier and is loaded with L‐arginine (L‐Arg), the HIF‐1α inhibitor lificiguat (YC‐1), and calcium phosphate (CaP). The nanomodulator exhibits TME responsiveness and near‐infrared (NIR) light‐controlled release capability. In a tumor‐specific acidic environment, the LYCHC NPs degrade progressively and release calcium ions (Ca^2+^), disrupting mitochondrial calcium homeostasis and collapsing the membrane potential. Concurrently, L‐Arg continuously generates NO via nitric oxide synthase, while YC‐1 effectively suppresses the HIF‐1α signalling pathway, providing dual intervention against tumor hypoxia. The combined action of NO and YC‐1 significantly improves oxygenation levels in the tumor region and, more importantly, alleviates the suppression of immune cell function caused by hypoxia, creating favourable conditions for the subsequent activation of the immune response. When exposed to 808 nm laser irradiation, LYCHC NPs demonstrate high‐efficiency photothermal conversion, which promotes the production of NO and the release of iron and copper ions. The released metal ions catalyse the production of substantial ROS via multi‐enzymatic activity, which synergises with NO to create a ROS/RNS storm. Notably, both ferroptosis and cuproptosis exhibit significant immunogenicity, leading to the release of abundant DAMPs, including extracellular release of CRT, HMGB1, and ATP. The DAMPs signaling molecules effectively promote the maturation of DCs and antigen presentation, thereby activating cytotoxic T lymphocytes and enhancing their infiltration into tumor tissues. LYCHC NPs can transform immunologically “cold” tumors into “hot” tumors, effectively remodeling the immunosuppressive microenvironment.

**SCHEME 1:**
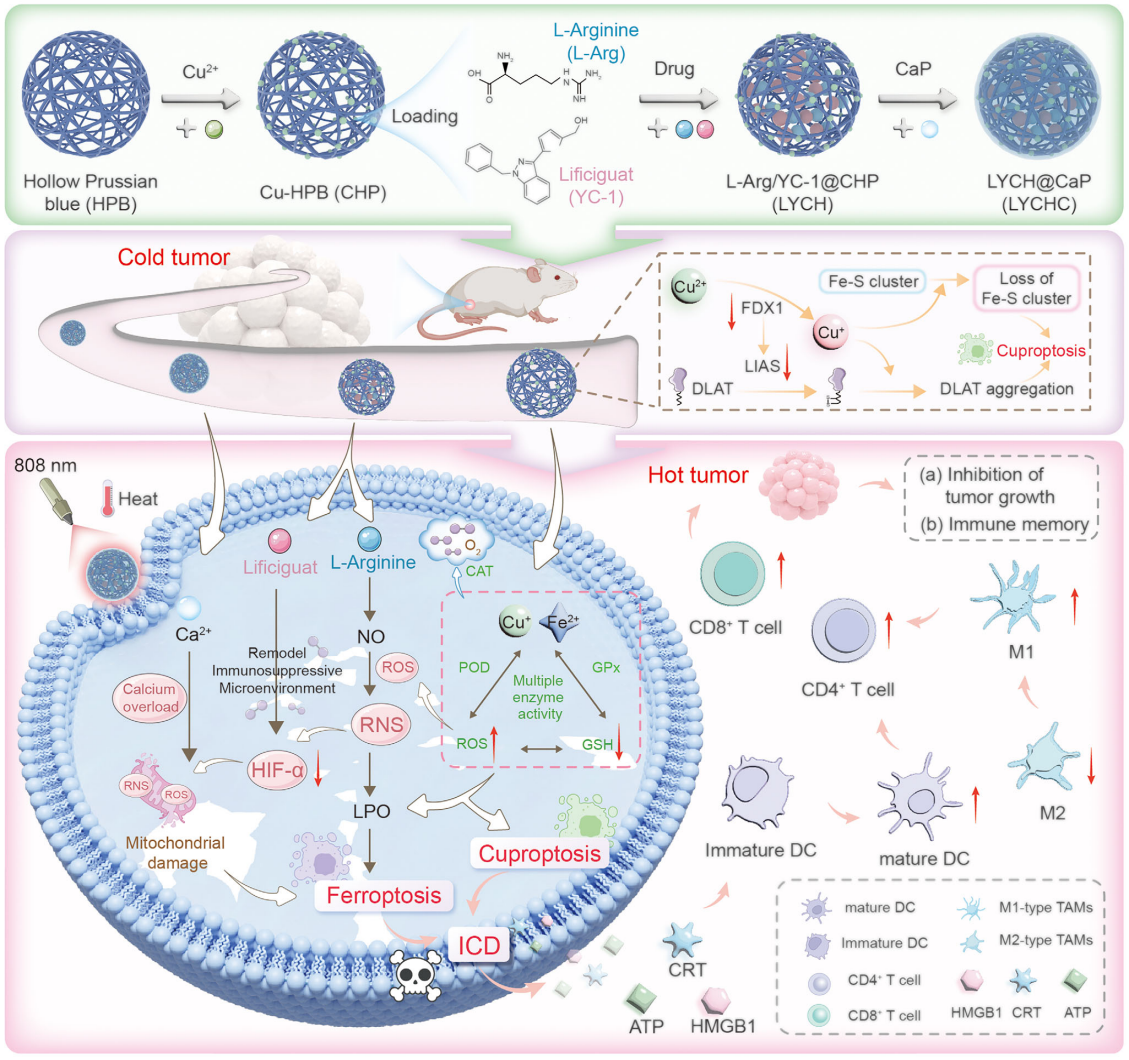
The multimodal nanomodulator for cascade ferroptosis/cuproptosis remodels the immunosuppressive tumor microenvironment.

## 2 Results and Discussion

### 2.1 Nanomodulator Synthesis and Acid‐Responsive Release

By selectively etching Prussian blue nanoparticles (PB NPs) to form a porous carrier and further introducing copper ions via ion exchange to adjust lattice parameters, the catalytic activity of the product is enhanced [54]. Acid‐responsive nanomodulator utilizes a hollow structure of CHP NPs as the core carrier, which simultaneously loads L‐Arg and YC‐1 for NO gas therapy and the alleviation of tumor hypoxia. The outer shell CaP confers acid‐responsive degradation properties for the targeted release of ions and drugs, and also induces mitochondrial calcium overload. LYCHC NPs are engineered to release their contents in a controlled manner when exposed to the acidic TME, laying the foundation for subsequent calcium overload induction, gas therapy, and metal ion‐mediated catalytic reactions. A simple, low‐toxicity hydrothermal method was employed to synthesize LYCHC NPs from PB NPs (Figure 1a).

**FIGURE 1:**
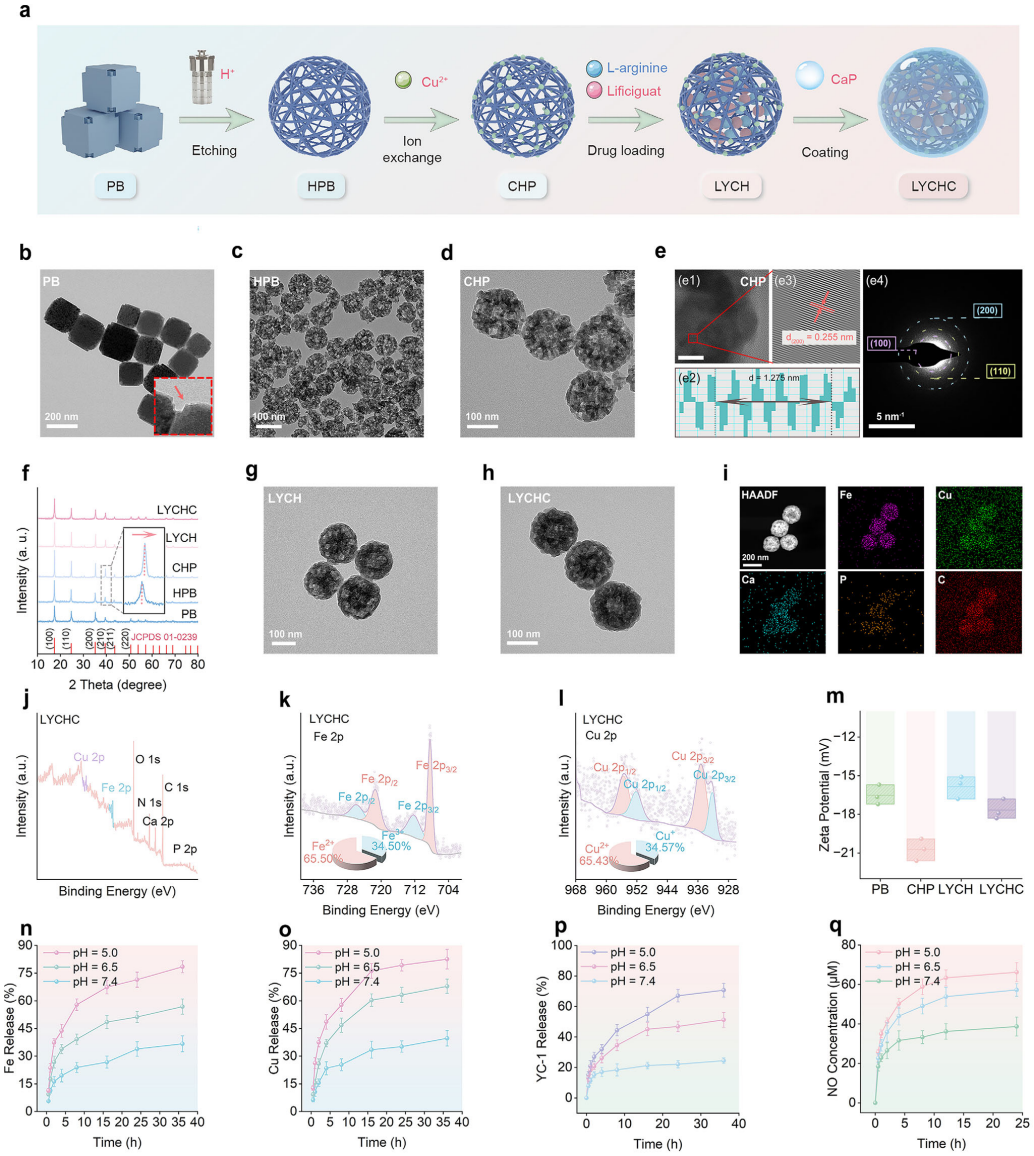
Nanomodulator synthesis and acid‐responsive release. (a) Schematic illustration for the synthetic process of LYCHC NPs. (b) TEM image of PB NPs. (c) TEM image of HPB NPs. (d) TEM image of CHP NPs. (e1) High‐resolution TEM image, (e2) interplanar spacing pattern, (e3) inverse fast Fourier transform micrograph, and (e4) SAED image of CHP NPs. (f) X‐ray diffraction patterns of PB, HPB, CHP, LYCH, and LYCHC NPs. (g) TEM image of LYCH NPs. (h) TEM image and (i) elemental mapping images of LYCHC NPs. (j) X‐ray photoelectron spectroscopy spectra of the survey region, (k) Fe 2p region, and (l) Cu 2p region of LYCHC NPs. (m) Zeta potentials of PB, CHP, LYCH, and LYCHC NPs. Regulation of (n) Fe ions, (o) Cu ions, and (p) YC‐1 release with different pH values (n = 3). (q) The concentration of NO produced by LYCHC NPs at different pH values (pH = 5.0, 6.5, and 7.4).

A transmission electron microscopy (TEM) image revealed distinct structural defects at the edges of PB NPs synthesized via the hydrothermal method, providing reaction sites for subsequent selective etching (Figure 1b). During hydrochloric acid etching, these coordination‐unsaturated defect sites dissolve preferentially due to their higher surface energy, causing the crystal to transform from a cubic structure into a spherical hollow structure rich in pores along its edges (Figure 1c). Hollow Prussian blue nanoparticles (HPB NPs) possess a unique morphology featuring both internal cavities and shell‐penetrating channels, making them an ideal carrier for efficient drug loading and surface functionalization. Copper elements were further incorporated into HPB NPs via ion exchange, successfully yielding CHP NPs. TEM image revealed that CHP NPs retain the spherical hollow structure of HPB NPs (Figure 1d). High‐resolution TEM images showed that LYCHC NPs exhibit an interplanar spacing of 0.255 nm, corresponding to the (200) plane of cubic PB NPs (Figure 1e1‐e2). The fast Fourier transform micrograph extracted from the brown‐marked region further reveals parallel stripe features corresponding to the (200) crystal plane (Figure 1e3). Diffraction rings corresponding to the (100), (110), and (200) crystal planes in the selected area electron diffraction pattern confirm the polycrystalline cubic phase structure of CHP NPs (Figure 1e4). X‐ray diffraction patterns reveal that the characteristic diffraction peaks of PB NPs perfectly match those of the standard card (JCPDS 01–0239) (Figure 1f). The resulting HPB NPs retain their intact crystal lattice structure after being etched with hydrochloric acid. The subsequent incorporation of copper ions induced a high‐angle shift in the diffraction peaks of the CHP NPs, originating from the replacement of certain Fe^2+^ (0.76 Å) by smaller Cu^2+^ (0.72 Å), which consequently reduced the lattice constant and contracted the interplanar distances [55].

L‐Arg and YC‐1 were loaded into the porous cavities of CHP NPs, constructing drug‐loaded L‐Arginine/lificiguat@Copper‐hollow Prussian blue nanoparticles (LYCH NPs). TEM image revealed significantly enhanced electron density in the central region of LYCH NPs compared to CHP NPs, indicating successful drug encapsulation (Figure 1g). Analysis of the specific surface area and pore size distribution further confirmed the loading of L‐Arg and YC‐1. Compared to CHP NPs (173.12 m^2^ g^−1^, 31.11 nm), the specific surface area of LYCH NPs decreased to 116.74 m^2^ g^−1^, and the pore size decreased to 15.71 nm (Figure S1). To enhance the specific responsiveness and biocompatibility of nanoparticles in the TME, a CaP shell was deposited onto the LYCH NPs surface via a biomimetic mineralization method, yielding LYCHC NPs (Figure 1h and Figure S2). Elemental mapping analysis and energy dispersive spectroscopy spectra reveal a uniform distribution of Fe, Cu, and Ca elements, with Ca elements colocalizing with P elements, confirming successful formation of the CaP shell (Figure 1i and Figure S3). X‐ray photoelectron spectroscopy analysis indicates Fe^2+^/Fe^3+^ and Cu^2+^/Cu^+^ ratios of 65.50%:34.50% and 65.43%:34.57% in LYCHC NPs (Figure 1j–l). The distinct valence state distribution of iron and copper ions facilitates the generation of ROS through enzymatic catalysis by LYCHC NPs within the TME, which is crucial for their catalytic therapeutic function. Zeta potential measurements revealed a decrease in negative charge from CHP NPs (−20.7 mV) to LYCH NPs (−15.8 mV), confirming drug loading of L‐Arg and YC‐1 (Figure 1m). The potential shift of LYCHC NPs to −17.7 mV aligns with CaP shell deposition characteristics, further validating the successful construction of the core‐shell structure. Dynamic light scattering size analysis indicated that LYCHC NPs had hydrated sizes of 160–165 nm, favorable for cellular uptake (Figure S4). Despite a polydisperse nature (PDI < 0.5), the hydrodynamic diameter remained stable within 160–170 nm without macroscopic aggregation over the test duration (Figure S5).

The TME responsiveness of LYCHC NPs was systematically evaluated through their release behavior under different pH conditions. The concentrations of iron and copper ions were determined using an iCAP 7400 inductively coupled plasma optical emission spectrometer (ICP‐OES). As shown in Figure 1n,o, both iron and copper ion release exhibited significant pH dependence. At pH = 7.4, the release rates were 36.70% and 39.7%, respectively, while at pH = 5.0, they increased to 78.50% and 82.6%. The differential ion release behavior under different conditions stems from the accelerated dissolution of the CaP shell under acidic conditions, exposing the CHP NPs and promoting ion release. YC‐1 release similarly exhibits pH‐responsive characteristics, increasing from 24.47% at pH = 7.4 to 70.70% at pH = 5.0 (Figure 1p and Figure S6) [56]. It is worth noting that YC‑1 achieves approximately 15.19% release at 0.5 h under acidic conditions, indicating that the drug can begin to be released within an early time window. This early release temporally aligns with the photothermal effect‑promoted ion release, providing a favorable pharmacokinetic basis for the subsequent intracellular generation of reactive oxygen species and reactive nitrogen species as well as the associated lipid peroxidation processes. Meanwhile, the sustained release over 24 h ensures a stable drug supply, and together with the continuous release of iron and copper ions, constitutes a spatiotemporally coordinated multi‑modal attack pattern. Meanwhile, under pH = 5.0 conditions, the concentration of NO generated from released L‐Arg reached 66.28 µM (Figure 1q and Figure S7). The systematic characterization from PB NPs precursors to the final composite architecture, coupled with the validation of acid‐responsive release behavior, confirms LYCHC NPs as a TME‐responsive drug delivery platform and establishes a critical initial step toward achieving avalanche effect‐triggered cascade ferroptosis and cuproptosis.

### 2.2 Photothermal Performance and Enzyme‐Mimicking Catalytic Activities

The nanomodulator LYCHC NPs combine highly efficient photothermal conversion capability with multi‐enzyme mimetic activity. Under NIR laser irradiation, the photothermal effect generated by the nanomodulator not only enables physical ablation of tumors but also serves as an external trigger switch to accelerate NO production and metal ion release, thereby synergistically enhancing catalytic performance. The multi‐enzyme activity simulated by LYCHC NPs aims to catalyze the production of abundant ROS and RNS within tumor cells. The oxidative stress storm creates the conditions necessary for irreversible LPO and the depletion of the antioxidant system.

UV−vis absorption spectra reveal pronounced concentration‐dependent absorption of LYCHC NPs in the NIR region, indicating their promising photothermal therapeutic potential (Figure S8). Compared to HPB NPs, copper‐doped CHP NPs, and the final LYCHC NPs exhibited significantly greater temperature elevation under 808 nm irradiation, confirming that copper doping effectively enhances the photothermal performance of nanoparticles (Figure 2a). Further studies revealed that the heating process of LYCHC NPs exhibits both power and concentration dependence (Figures S9 and S10). After four laser switching cycles, the nanoparticles maintained stable heating capability, demonstrating excellent photothermal stability (Figure 2b). Calculations yielded a photothermal conversion efficiency of 40.63% for LYCHC NPs, indicating their highly efficient photothermal conversion performance (Figure S11). In vivo experiments further validated the application potential of LYCHC NPs. Following injection of LYCHC NPs, the temperature in the tumor region of mice elevated to over 48°C within 10 min under 808 nm irradiation, sufficient to effectively induce tumor cell ablation (Figure 2c,d). Local hyperthermia not only directly kills tumor cells but may also enhance the ability of LYCHC NPs to promote the release of tumor‐associated antigens, thereby creating favorable conditions for activating anti‐tumor immunity.

**FIGURE 2:**
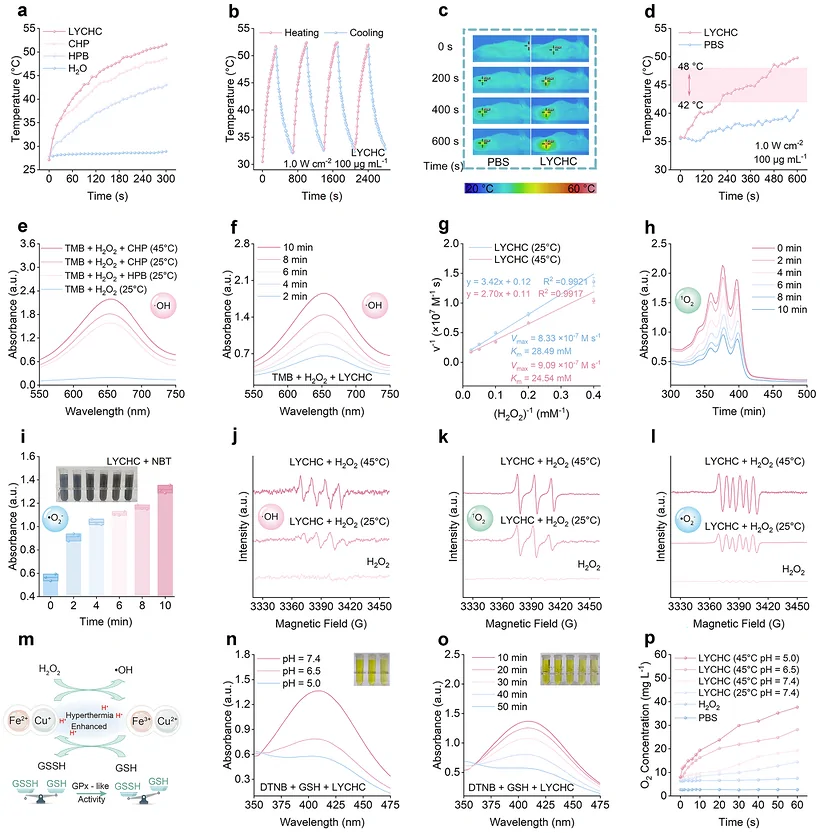
Photothermal performance and enzyme‐mimicking catalytic activities. (a) Temperature‐time curves of H_2_O, HPB, CHP, and LYCHC NPs solutions under NIR irradiation (1.0 W cm^−2^). (b) Temperature curve of LYCHC NPs under NIR irradiation for four laser on/off cycles (1.0 W cm^−2^, 100 µg mL^−1^). (c) In vivo thermal imaging of mice in the tumor region. (d) In vivo photothermal capability of LYCHC NPs. (e) UV−Vis absorption spectra of oxidized TMB recorded under different conditions. (f) Time‐dependent absorbance spectra of oxidized TMB in the presence of H_2_O_2_ and LYCHC NPs. (g) Lineweaver–Burk plotting for POD‐like activity of LYCHC NPs. Changes in the UV–vis absorption spectra of (h) ABDA and (i) NBT at different reaction times. ESR spectra of (j)·OH, (k) ^1^O_2_, and (l) ·O_2_^−^ captured after different treatments. (m) Schematic representation of enzyme activity and GPx activities of LYCHC NPs. (n‐o) The GSH depletion capacity of LYCHC NPs. (p) The concentration of O_2_ in different solutions.

Beyond the immune activation effect, the core killing mechanism of LYCHC NPs lies in their exceptional nanozyme activity, which triggers an “avalanche effect” cascade reaction within tumor cells. The ability of LYCHC NPs to catalyze H_2_O_2_ to produce ·OH was evaluated using the 3,3',5,5'‐tetramethylbenzidine (TMB) colorimetric assay. As shown in Figure 2e, the absorbance of CHP NPs at 652 nm was significantly higher than that of HPB NPs. When the solution temperature was elevated to 45°C, the ability of CHP NPs to generate ·OH further increased, indicating that the copper ion doping strategy effectively enhanced their catalytic performance. As shown in Figure 2f and Figure S12, the oxidation process of TMB in the presence of LYCHC NPs exhibits distinct time‐dependent and concentration‐dependent characteristics. Michaelis‐Menten curves indicate that the maximum reaction rate of LYCHC NPs at 45°C is higher than that at 25°C (Figure S13). The kinetic parameters obtained from Lineweaver‐Burk fitting indicate that as the temperature increased from 25°C to 45°C, the *K*_m_ value decreased from 28.49 to 24.54 mM, while the *V*_max_ increased from 8.33 × 10^−7^ M s^−1^ to 9.09 × 10^−7^ M s^−1^ (Figure 2g). The decrease in *K*_m_ and increase in *V*_max_ collectively confirm that the photothermal effect effectively enhances the catalytic efficiency and substrate affinity of LYCHC NPs. During photothermal therapy, the ROS‐generating capacity of LYCHC NPs will be amplified synchronously, thereby achieving synergistic effects between photothermal and enzymatic activities. Additionally, LYCHC NPs can also catalyze the production of other types of ROS. Using 9,10‐anthracenediyl‐bis(methylene)dimalonic acid (ABDA) as a ^1^O_2_ probe, its absorption peak intensity decreased significantly over time, indicating that LYCHC NPs can effectively generate ^1^O_2_ (Figure 2h). The generation of ·O_2_^−^ was detected using nitroblue tetrazolium chloride (NBT). The absorbance of the solution at 560 nm increased over time, confirming that LYCHC NPs can efficiently produce ·O_2_^−^ (Figure 2i). Electron spin resonance (ESR) analysis further revealed the distinct types of ROS generated by LYCHC NPs. Using 5,5‐dimethyl‐1‐pyrroline N‐oxide (DMPO) as a capture agent, a distinct ·OH signal was detectable at 25°C, with a significant enhancement observed at 45°C, indicating that the photothermal effect promotes Fe^2+^/Cu^+^‐mediated ·OH generation (Figure 2j). Using 2,2,6,6‐tetramethylpiperidine (TEMP) as the capture agent, the ^1^O_2_ signal intensity at 45°C was higher than that at 25°C (Figure 2k). A similar trend was observed when DMPO was used to scavenge ·O_2_^−^ (Figure 2l). ESR analysis results indicate that LYCHC NPs can simultaneously generate multiple ROS, including ·OH, ^1^O_2_, and ·O_2_^−^. The synergistic burst of multiple ROS types not only directly induces LPO but also lays the groundwork for subsequent reactions with NO to generate RNS and activate ferroptosis and cuproptosis pathways.

In the acidic TME, LYCHC NPs function as a pH‐responsive nanomodulator, releasing Fe ions and Cu ions. Fe^2+^ and Cu^+^ predominantly mediate enzyme activity, catalyzing the conversion of H_2_O_2_ into ·OH, ^1^O_2_, and ·O_2_^−^. Meanwhile, Cu^2+^ and Fe^3+^ mimic glutathione peroxidase (GPx) function, synergistically depleting GSH. The Cu^2+^/Cu^+^ and Fe^2+^/Fe^3+^ dual‐cycle mechanism established by LYCHC NPs enables continuous regeneration of catalytic substrates, forming a self‐sustaining catalytic cycle (Figure 2m). GSH depletion experiments revealed that LYCHC NPs exhibited significantly higher consumption rates under acidic conditions compared to physiological pH, achieving rapid and sustained GSH depletion within 50 min. Depletion of GSH disrupts intracellular antioxidant defenses, promoting ROS accumulation and LPO (Figure 2n,o). Furthermore, dissolved oxygen measurements indicate that LYCHC NPs induce an increase in oxygen (O_2_) concentration under NIR irradiation, with a more pronounced effect in acidic environments. The results in Figure 2p confirm the ability of LYCHC NPs to alleviate tumor hypoxia. The O_2_‐generating capacity of LYCHC NPs synergistically enhances the function of the HIF‐1α inhibitor YC‐1 loaded within the nanoparticles. The former increases O_2_ supply while the latter suppresses hypoxia signaling pathways, collectively dismantling the tumor's hypoxic adaptation mechanisms. LYCHC NPs, leveraging their exceptional catalytic properties, can induce bursts of ROS within tumor cells and disrupt the antioxidant defense system while simultaneously improving the tumor‐suppressive microenvironment, thereby providing a foundation for synergistic antitumor strategies. In summary, through synergistic photothermal conversion and multi‐enzyme‐mimetic activity strategies, LYCHC NPs establish a highly oxidative microenvironment at the tumor site, providing the core driving force for inducing ferroptosis and cuproptosis within the tumor.

### 2.3 Cellular Internalization and Reactive Oxygen Species Burst

The therapeutic strategy of LYCHC NPs relies on their efficient uptake by tumor cells and subsequent disintegration within the acidic environment of intracellular lysosomes. By inducing multiple stressors, including intracellular calcium overload and ROS storms, LYCHC NPs effectively disrupt cellular homeostasis, thereby laying the groundwork for the realization of ICD. Given the outstanding catalytic and photothermal properties of LYCHC NPs, their antitumor efficacy and underlying mechanisms are highly anticipated in vitro.

The CT26 colon carcinoma cell line was employed to elucidate how LYCHC NPs achieve highly efficient and specific killing by triggering an intracellular “avalanche effect” of multiple stresses (Figure 3a). In vitro cytotoxicity assays demonstrated that LYCHC NPs exhibited no significant toxicity toward normal L929 fibroblasts even at high concentrations up to 400 µg mL^−1^, with cell viability remaining above 85%, in stark contrast to the pronounced concentration‐dependent killing effect observed in CT26 tumor cells (Figure 3b,c). Notably, the LYCHC + NIR group elicited the most potent cytotoxic effect, with cell viability dropping below 60% at 100 µg mL^−1^. The results of the cytotoxicity experiments not only preliminarily validate the photothermal synergistic enhancement of LYCHC NPs but also provide primary evidence for their capacity to trigger tumor‐specific avalanche‐like cascade reactions. FITC‐labeled LYCHC NPs were co‐incubated with CT26 cells. Confocal laser scanning microscopy (CLSM) images and line scans revealed a sustained increase in green fluorescence signals over extended incubation periods, indicating efficient cellular uptake and time‐dependent intracellular accumulation of LYCHC NPs (Figure 3d). The efficient internalization of cells is a prerequisite for the functioning of LYCHC NPs.

**FIGURE 3:**
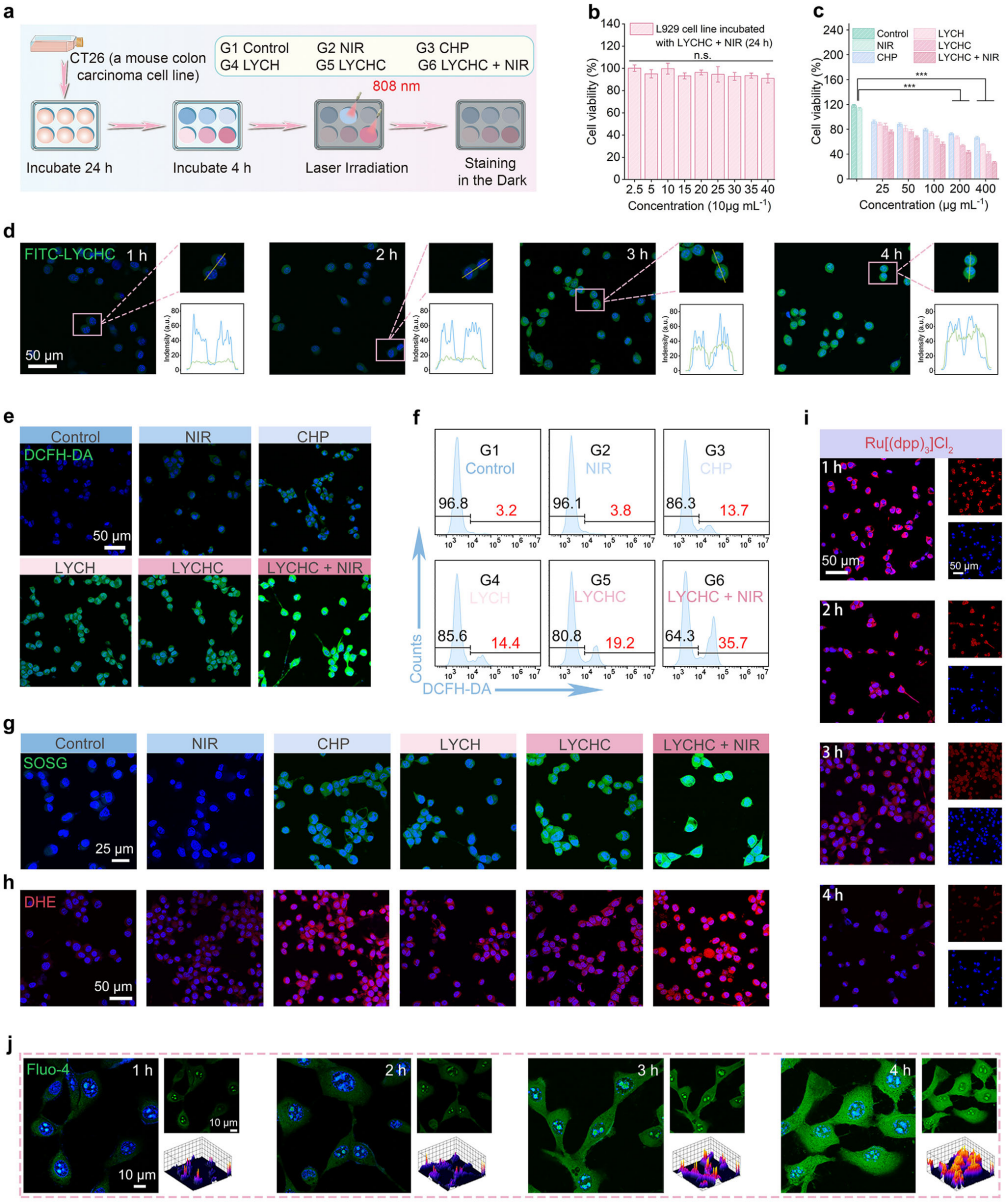
Cellular internalization and reactive oxygen species burst. (a) Therapeutic schematic diagram of CT26 cells. Relative cell viability of (b) L929 cells and (c) CT26 cells after different treatments (n = 3, mean ± SD). (d) CLSM images and corresponding line scan curves of CT26 cells incubated with FITC‐LYCHC NPs at different time intervals. (e) CLSM images and (f) flow cytometry were used to analyze the intracellular ROS levels of CT26 cells after different treatments. CLSM images were used to observe the levels of (g) ^1^O_2_ and (h) ·O_2_^−^ within cells. The accumulation of (i) O_2_ and (j) Ca^2+^ within cells at different times. (G1: Control; G2: NIR; G3: CHP; G4: LYCH; G5: LYCHC; G6: LYCHC + NIR; ^*^p < 0.05, ^**^p < 0.01, ^***^p < 0.001, and *ns*: not significant).

Following cellular entry, LYCHC NPs first triggered two rapid initial stress responses: oxidative stress and calcium homeostasis disruption. The oxidative stress mechanism triggered by LYCHC NPs was investigated in detail. Detection of total intracellular ROS levels revealed that both fluorescence signals and flow cytometric quantification in the LYCHC + NIR group were most pronounced, indicating that the treatment induced a dramatic ROS burst (Figure 3e,f). To further characterize ROS species, specific detection of ^1^O_2_ and ·O_2_^−^ was performed. As shown in Figure 3g, the LYCHC + NIR group exhibits the strongest ^1^O_2_ signal in the singlet oxygen sensor green (SOSG) staining, primarily attributed to the photothermal effect and the highly efficient catalytic properties of iron and copper ions. Meanwhile, dihydroethidium (DHE) staining in Figure 3h revealed significantly enhanced fluorescence intensity in the LYCHC + NIR group compared to the control group, indicating elevated intracellular ·O_2_^−^ levels following this treatment. Given that the mitochondrial electron transport chain serves as the primary source of intracellular ·O_2_^−^, LYCHC + NIR treatment may induce mitochondrial functional stress or early damage. In Figure 3i, O_2_ concentration‐sensitive probes [Ru(dpp)_3_]Cl_2_ were employed to monitor intracellular O_2_ kinetics. After 4 h of LYCHC NPs treatment, the intracellular fluorescence intensity significantly decreased, indicating substantial production of O_2_ within the cells. Alleviating hypoxia in the TME is a key link for LYCHC NPs to remodel the immunosuppressive microenvironment. The catalase‐like activity of LYCHC NPs enables the decomposition of excessively accumulated H_2_O_2_ within tumors into O_2_, thereby directly alleviating tumor hypoxia stress. Additionally, O_2_ promotes L‐Arg metabolism to generate NO and improves oxygen delivery by dilating tumor blood vessels, thereby synergistically inhibiting the HIF‐1α pathway with YC‐1. LYCHC NPs not only significantly reduced tumor hypoxia but also disrupted HIF‐1α‐driven tumor metabolic reprogramming. Beyond oxidative stress, calcium homeostasis disruption induced by LYCHC NPs represents another key mechanism. Intracellular Ca^2+^ level imaging revealed a time‐dependent increase in cytoplasmic fluorescence intensity, confirming calcium overload triggered by rapid degradation of the outer CaP shell in the acidic TME (Figure 3j). Disruption of calcium homeostasis directly leads to mitochondrial membrane potential collapse, exacerbating cellular energy metabolism dysfunction [57].

Overall, following internalization by CT26 tumor cells, LYCHC NPs successfully triggered the initial intracellular stress signaling networks through the synergistic activation of a NIR laser and the acidic TME. By inducing mitochondrial calcium overload and generating ROS, they ultimately activate both ferroptosis and cuproptosis pathways, laying a crucial foundation for remodeling the immunosuppressive microenvironment.

### 2.4 Activate the Dual Immunogenic Cell Death Pathways of Ferroptosis and Cuproptosis

The nanomodulator LYCHC NPs are engineered to simultaneously activate both ferroptosis and cuproptosis, two emerging cell death pathways. Intracellularly released iron and copper ions from LYCHC NPs catalyze extensive LPO while depleting the key antioxidant molecule GSH and directly inhibiting the activity of crucial enzymes such as GPX4. Furthermore, copper ions specifically target acylated mitochondrial enzymes, disrupting the tricarboxylic acid cycle [58]. Through multiple synergistic pathways that trigger cellular metabolic collapse and membrane system damage, LYCHC NPs ultimately strongly induce high ICD. A key mechanism of action for LYCHC NPs lies in inducing intense oxidative/nitrosative stress. Detection using specific fluorescent probes revealed significantly elevated levels of NO and peroxynitrite (ONOO^−^) in LYCHC + NIR‐treated CT26 cells, indicating that L‐Arg was successfully metabolized into NO (Figure 4a). The ·O_2_^−^ and NO reactions induced by LYCHC NPs generate ONOO^−^, which possesses strong oxidizing and nitrosating capabilities. ONOO^−^, as a key RNS, exhibits significantly higher toxicity than most free radicals, including ·OH, and participates in various physiological and pathological processes such as LPO, protein nitrosylation, and oxidative DNA damage [59]. Due to its high reactivity, ONOO^−^ rapidly diffuses and targets the cellular powerhouse (mitochondria), becoming the primary factor causing mitochondrial dysfunction. Bio‐TEM images revealed that the LYCHC + NIR group exhibited pathological alterations, including marked mitochondrial shrinkage, increased membrane density, and reduced cristae structure, consistent with the typical mitochondrial morphology associated with ferroptosis and cuproptosis (Figure 4b). The CLSM images in Figure S14 further revealed that LYCHC + NIR treatment caused severe collapse of the intracellular mitochondrial membrane potential, manifested by significantly enhanced green fluorescence (monomers) and reduced red fluorescence (polymers). The loss of mitochondrial membrane potential signifies an imbalance in energy metabolism and a vicious cycle of ROS production, exacerbating oxidative damage within the cell.

**FIGURE 4:**
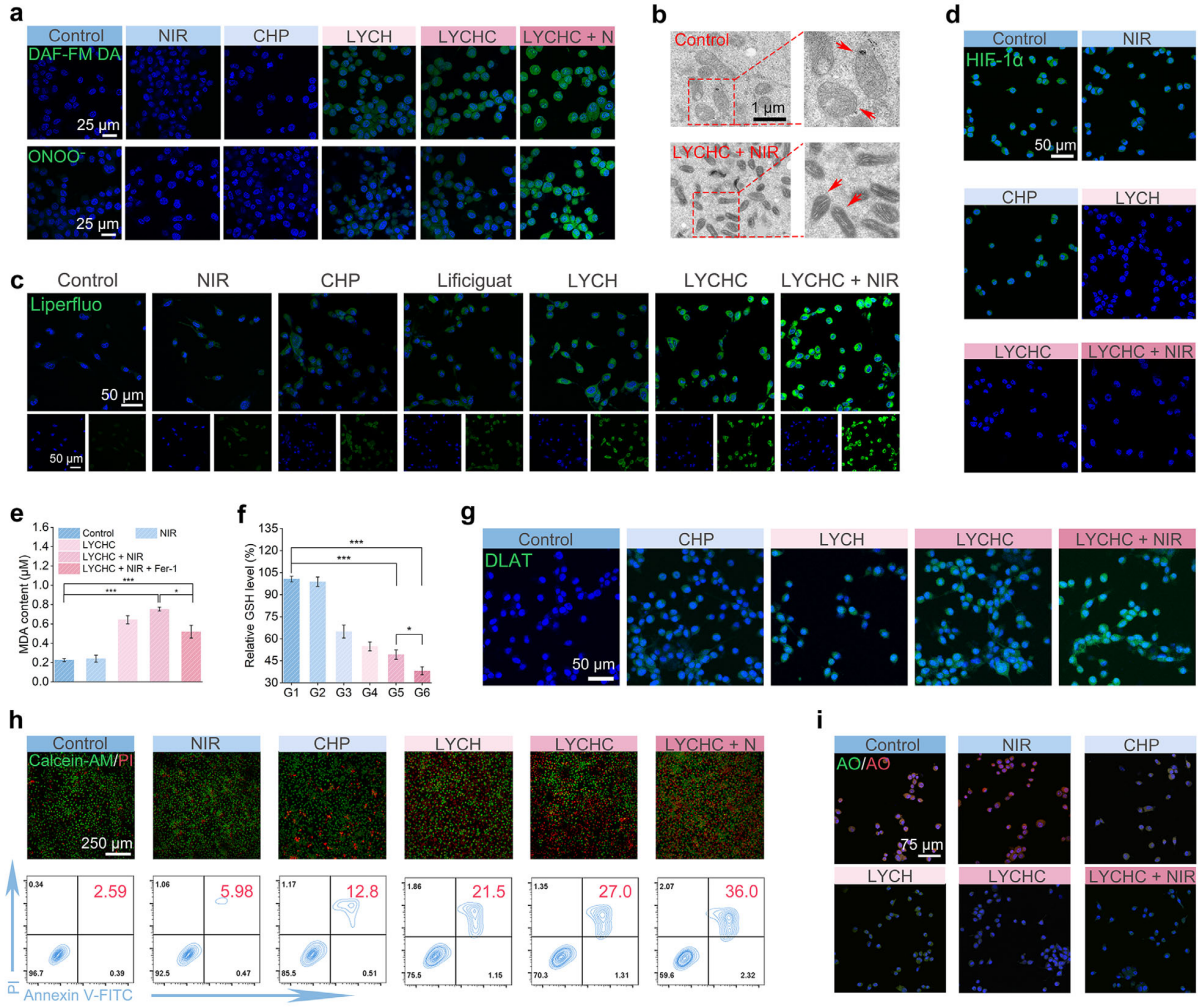
Activate the dual immunogenic cell death pathways of ferroptosis and cuproptosis. (a) CLSM images of CT26 tumor cells stained with NO probe and ONOO^−^ probe after different treatments. (b) Bio‐TEM images of CT26 cells in the control group and LYCHC + NIR group. CLSM images stained with (c) lipofuscin probe and (d) HIF‐1α probe after different treatments. Evaluation of (e) MDA and (f) GSH levels within CT26 cells (n = 3, mean ± SD). (g) Immunofluorescence images of CT26 cells stained with anti‐DLAT antibody. (h) CLSM images of apoptosis conditions revealed by Calcein AM/PI probes and flow cytometric apoptosis analysis of Annexin V‐FITC/PI‐stained cells after different treatments. (i) CLSM images of CT26 tumor cells stained with the AO probe. (G1: Control; G2: NIR; G3: CHP; G4: LYCH; G5: LYCHC; G6: LYCHC + NIR; ^*^p < 0.05, ^**^p < 0.01, ^***^p < 0.001, and *ns*: not significant).

Dysfunctional mitochondria not only promote ROS production, but the peroxidation of their own membrane lipids also triggers chain reactions, ultimately inducing more severe oxidative damage. As demonstrated by liperfluo probe results, the LYCHC + NIR group induced the most significant elevation in LPO levels. The YC‐1 group elicited only a faint fluorescent signal, highlighting the synergistic role of metal ions and RNS in driving LPO (Figure 4c). To further dissect the individual contributions of L‑Arg and YC‑1, MTT assays were performed on CT26 cells treated with single‑agent or dual‑agent loaded nanoparticles. As shown in Figure S15, free L‑Arg or free YC‑1 alone exhibited negligible cytotoxicity with cell viabilities of 95.55% and 97.70%, respectively, indicating that the nanocarrier is essential for efficient intracellular delivery. In contrast, CHP NPs alone reduced cell viability due to iron/copper ion‑induced oxidative stress. The single‑agent groups, CHP + L‑Arg and CHP + YC‑1, decreased cell viability to 81.81% and 85.15%, respectively, confirming that L‑Arg‑derived NO production and YC‑1‑mediated HIF‑1α inhibition each independently suppress tumor cell survival when delivered by CHP NPs. Notably, the co‑loaded LYCH group further reduced viability to 67.01%, which was significantly lower than that achieved by either single‑agent group. This enhanced efficacy exceeds a mere additive effect, revealing true synergy between L‑Arg‑derived NO production and YC‑1‑mediated HIF‑1α blockade in driving tumor cell death. Immunofluorescence results confirmed that LYCHC + NIR treatment effectively downregulated HIF‐1α expression, suggesting it enhances cellular sensitivity to oxidative damage by alleviating tumor hypoxia (Figure 4d). The individual effect of YC‑1 on HIF‑1α suppression was further evaluated. Figure S16 shows that CHP + YC‑1 treatment produced a more pronounced decrease in HIF‑1α fluorescence intensity compared to CHP + L‑Arg, while the LYCH group exhibited the weakest signal, demonstrating a synergistic inhibition of HIF‑1α by L‑Arg‑derived NO and YC‑1. LYCHC NPs induced a significant increase in malondialdehyde (MDA) levels within CT26 cells. Ferroptosis inhibitor Ferrostatin‐1 reversed the MDA elevation induced by LYCHC NPs, confirming the critical role of the ferroptosis pathway (Figure 4e). Concurrently, intracellular levels of the primary antioxidant molecule GSH were markedly depleted, further confirming successful activation of the ferroptosis pathway (Figure 4f). Cuproptosis is a form of cell death mediated by mitochondrial metabolic enzyme abnormalities, characterized by excessive copper ions, accumulation of lipoylated DLAT, and loss of Fe‐S cluster proteins. Immunofluorescence imaging revealed marked DLAT accumulation within cells following LYCHC NPs addition, in stark contrast to the control group (Figure 4g and Figure S17). The reductase ferredoxin 1 (FDX1) reduces Cu^2+^ to the more toxic Cu^+^ and regulates the sulfhydryl modification of DLAT. DLAT present in mitochondria undergoes oligomerization and increased insolubility after binding with Cu^+^, inducing an abnormal tricarboxylic acid cycle, which is the key point for the occurrence of cuproptosis [58]. Western blot analysis of key executioner molecules provided direct evidence for the synergistic interplay. As shown in Figure S18, GPX4 expression was slightly downregulated in the CHP + L‑Arg group and more pronounced in the CHP + YC‑1 group. Critically, the LYCH group exhibited substantially lower GPX4 and DLAT levels than either single‑agent group, and the LYCHC+NIR group achieved near‑complete knockdown. YC‑1‑mediated HIF‑1α inhibition dismantles the cellular defense foundation, allowing NO‑driven oxidative stress to effectively suppress both ferroptosis and cuproptosis regulators.

LYCHC NPs ingeniously synchronously activate both ferroptosis and cuproptosis by inducing mitochondrial dysfunction and ROS/RNS storms. The synergistic induction of two emerging cell death pathways constitutes the core foundation of LYCHC NPs' potent, multi‐targeted tumor cell killing capacity. Ultimately, the multi‐pathway, multi‐target synergistic attack resulted in widespread tumor cell death. Calcein‐AM/PI staining revealed extensive cell death in the LYCHC + NIR group. Flow cytometry further confirmed that LYCHC NPs induced a significant increase in the proportion of late apoptosis and necrosis in tumor cells (Figure 4h). Acridine orange (AO) staining also revealed characteristic nuclear damage patterns (Figure 4i). Collectively, the interplay between ferroptosis and cuproptosis operates as a synergistic amplification mechanism rather than a simple additive effect. Fe^2+^ mediated multi‐enzyme mimetic activity initiates LPO and suppresses GPX4, while Cu^2+^ accelerates oxidative damage through Fenton‐like reactivity and directly targets mitochondrial DLAT to trigger cuproptosis. The synergy is achieved through coordinated GSH depletion, where Cu^2+^ chelation and Fe^2+^ induced oxidative stress jointly dismantle the antioxidant barrier. Concurrently, cuproptosis‐mediated mitochondrial dysfunction amplifies Fe^2+^ initiated LPO, establishing a positive feedback loop. NO further bridges these pathways by generating cytotoxic RNS and inactivating antioxidant enzymes. The dual pathway coactivation enhances tumor cell killing and amplifies ICD for remodeling the immunosuppressive microenvironment.

### 2.5 Immunogenic Cell Death Activation and Transcriptomic Profiling

Through multiple synergistic mechanisms, LYCHC NPs effectively reverse immunosuppression, induce ICD, activate DCs maturation, promote T cell infiltration, ultimately leading to primary tumor suppression and the development of systemic immune memory. Immunofluorescence results showed that in the LYCHC + NIR group, CRT was significantly exposed to the surface of CT26 cell membranes, forming a distinct outline, which is a sign of effective “eat me” signal activation (Figure 5a). Concurrently, HMGB1 nuclear fluorescence intensity markedly diminished, suggesting the key DAMPs were being released from the nucleus into the extracellular space (Figure 5b). Extracellular HMGB1 enhances innate immune responses by binding to receptors such as TLR4 on the surface of DCs and macrophages, thereby promoting proinflammatory cytokine secretion and laying the groundwork for subsequent adaptive immune activation. As shown in Figure 5c, compared to the Control group, LYCHC NPs treatment reduced intracellular ATP levels by 50.81%, while the LYCHC + NIR group further decreased intracellular ATP by 63.04%. The drastic depletion of intracellular ATP signifies the collapse of mitochondrial energy metabolism, a process typically accompanied by the release of ATP from dying cells into the extracellular space. Extracellular ATP serves as a crucial “find me” signal, recruiting antigen‐presenting cells and promoting their maturation, thereby participating in the regulation of the immunosuppressive microenvironment [60].

**FIGURE 5:**
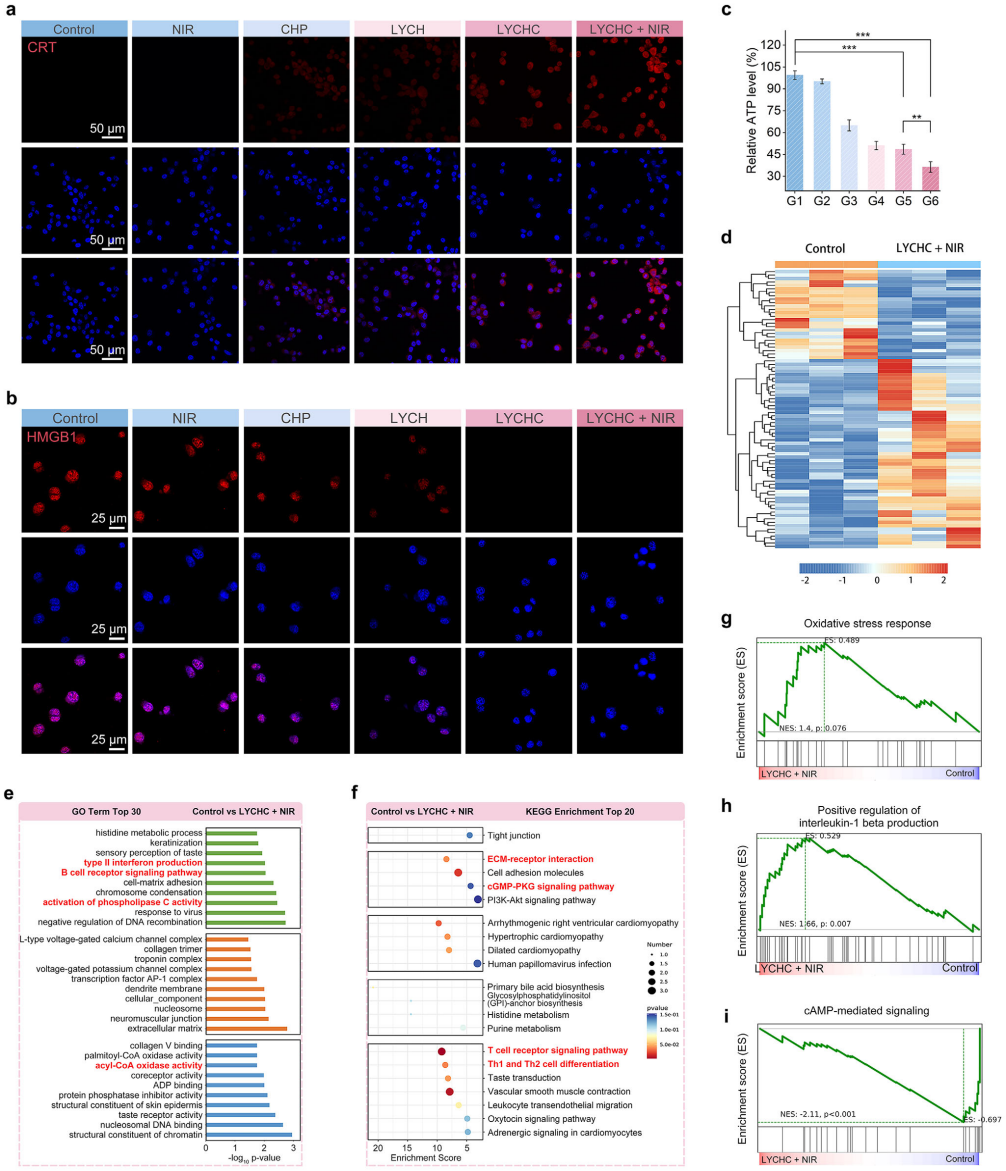
Immunogenic cell death activation and transcriptomic profiling. CLSM images of (a) CRT expression and (b) HMGB1 release of CT26 cells after different treatments. (c) Intracellular ATP levels after diverse treatments (n = 3). (d) Volcano map of genes expressed differentially in the LYCHC + NIR group. (e) KEGG enrichment analysis and (f) GO enrichment analysis of the differentially expressed genes in the LYCHC + NIR group. (g‐i) GSEA enrichment analysis of the differentially expressed genes in the LYCHC + NIR group. (G1: Control; G2: NIR; G3: CHP; G4: LYCH; G5: LYCHC; G6: LYCHC + NIR; ^*^p < 0.05, ^**^p < 0.01, ^***^p < 0.001, and *ns*: not significant).

To systematically reveal the molecular blueprint governing immune microenvironment regulation at the genomic scale, treated CT26 cells underwent full transcriptome sequencing analysis. Volcano plots revealed extensively differentially expressed genes in CT26 cells following LYCHC + NIR treatment (Figure 5d). Gene Ontology (GO) analysis revealed that differentially expressed genes were significantly enriched in multiple immune‐related pathways, including the type II interferon production signaling pathway, B cell receptor signaling pathway, activation of phospholipase C activity signaling pathway, and acyl‐CoA oxidase activity signaling pathway (Figure 5e). The activation of the acyl‐CoA oxidase activity pathway suggests that lipid metabolism reprogramming may play a crucial role in the LYCHC NPs‐induced ICD process. Kyoto Encyclopedia of Genes and Genomes (KEGG) enrichment analysis further revealed significant enrichment of the cGMP‐PKG signaling pathway, indicating that LYCHC NPs may activate PKG by upregulating cGMP levels, thereby suppressing HIF‐1α stability, alleviating tumor hypoxia, and enhancing immune cell function (Figure 5f). Western blot analysis was employed to validate the pathways predicted by transcriptomic analysis. As shown in Figure S19, LYCHC + NIR treatment significantly increased PKG phosphorylation and reduced HIF‐1α expression, effects that were effectively reversed by the PKG inhibitor KT5823. Moreover, the significant enrichment of T cell receptor signaling and Th1/Th2 cell differentiation pathways directly links the therapeutic effect to T cell‐mediated core antitumor immune responses [61]. Effective differentiation of naive T cells into antitumor effector Th1 subsets was observed upon treatment with LYCHC NPs, along with the establishment of long‐term immunological memory. Gene set enrichment analysis (GSEA) further revealed that differentially expressed genes significantly clustered in the “Oxidative stress response” and “Positive regulation of interleukin‐1 beta production” pathways. As a key product of inflammasome activation, IL‐1β effectively promotes T cell activation and proliferation, enhancing immune killing capacity (Figure 5g,h). Concurrently, the “cAMP‐mediated signaling” pathway was also significantly activated, which synergistically regulates cellular metabolism and immune responses through cGMP‐PKG (Figure 5i). The transcriptomic findings reveal that LYCHC NPs simultaneously improve the TME through pathways like cGMP‐PKG and directly enhance immune cell killing capacity by promoting key cytokines such as IL‐1β. Ultimately, they regulate T cell differentiation to drive effective antitumor immunity. In summary, LYCHC NPs synergistically induce ICD through multiple mechanisms under 808 nm NIR laser regulation while remodeling the immunosuppressive microenvironment. Compared to traditional single‐mode ICD inducers, LYCHC NPs demonstrate significant advantages by simultaneously activating multiple pathways of innate and adaptive immunity. The strategy of LYCHC + NIR not only effectively controls primary tumors through synergistic induction of novel ICD mechanisms like ferroptosis and cuproptosis but also establishes systemic immune memory, offering a forward‐looking therapeutic approach for suppressing tumor recurrence and distant metastasis.

### 2.6 Primary Tumor Eradication via Immunosuppressive Microenvironment Remodeling

By using the enhanced permeability and retention (EPR) effect for tumor accumulation, LYCHC NPs enable precise intratumoral drug release and multi‐enzyme activity, thereby effectively inhibiting primary tumor growth and remodeling the immunosuppressive microenvironment via hypoxia mitigation. To simulate the characteristics of clinically metastatic tumors, the primary and distant tumor models were established to evaluate the direct cytotoxic effects of LYCHC NPs on primary tumors and their inhibitory effects on distant tumors (Figure 6a). Biodistribution analysis at 48 h post‐injection demonstrated that Fe and Cu elements accumulated predominantly in tumor tissues via the EPR effect, while maintaining relatively low distribution levels in major organs including heart, liver, spleen, lung, and kidney, with progressive clearance over time (Figure 6b,c). Under the weakly acidic conditions of the TME, the CaP shell degraded, facilitating the release of Fe ions and Cu ions from LYCHC NPs. The specific release of ions is a prerequisite for LYCHC NPs to subsequently induce ferroptosis and cuproptosis synergistically within tumor cells. Pharmacokinetic analysis revealed that the plasma concentration‐time curve of LYCHC NPs conformed to a two‐compartment model, with a distribution half‐life τ_1/2(α)_ of 0.21 h and an elimination half‐life τ_1/2(β)_ of 5.21 h (Figure 6d). The prolonged circulation within the body ensures the retention of LYCHC NPs in the bloodstream, facilitating their efficient accumulation at tumor sites through EPR effects. The clearance rate curve further reveals its characteristic biphasic clearance pattern (Figure 6e). In vivo near‑infrared fluorescence imaging Figure S20 showed that Cy5‑labeled LYCHC NPs gradually accumulated in the tumor site, with fluorescence signals detectable at 8 h post‑injection, peaking at 12 h, and remaining elevated at 48 h. In contrast, fluorescence in normal tissues progressively declined over time. The selective and prolonged tumor retention confirmed that LYCHC NPs accumulated via the EPR effect, providing the basis for subsequent acid‑responsive drug release and photothermal therapy.

**FIGURE 6:**
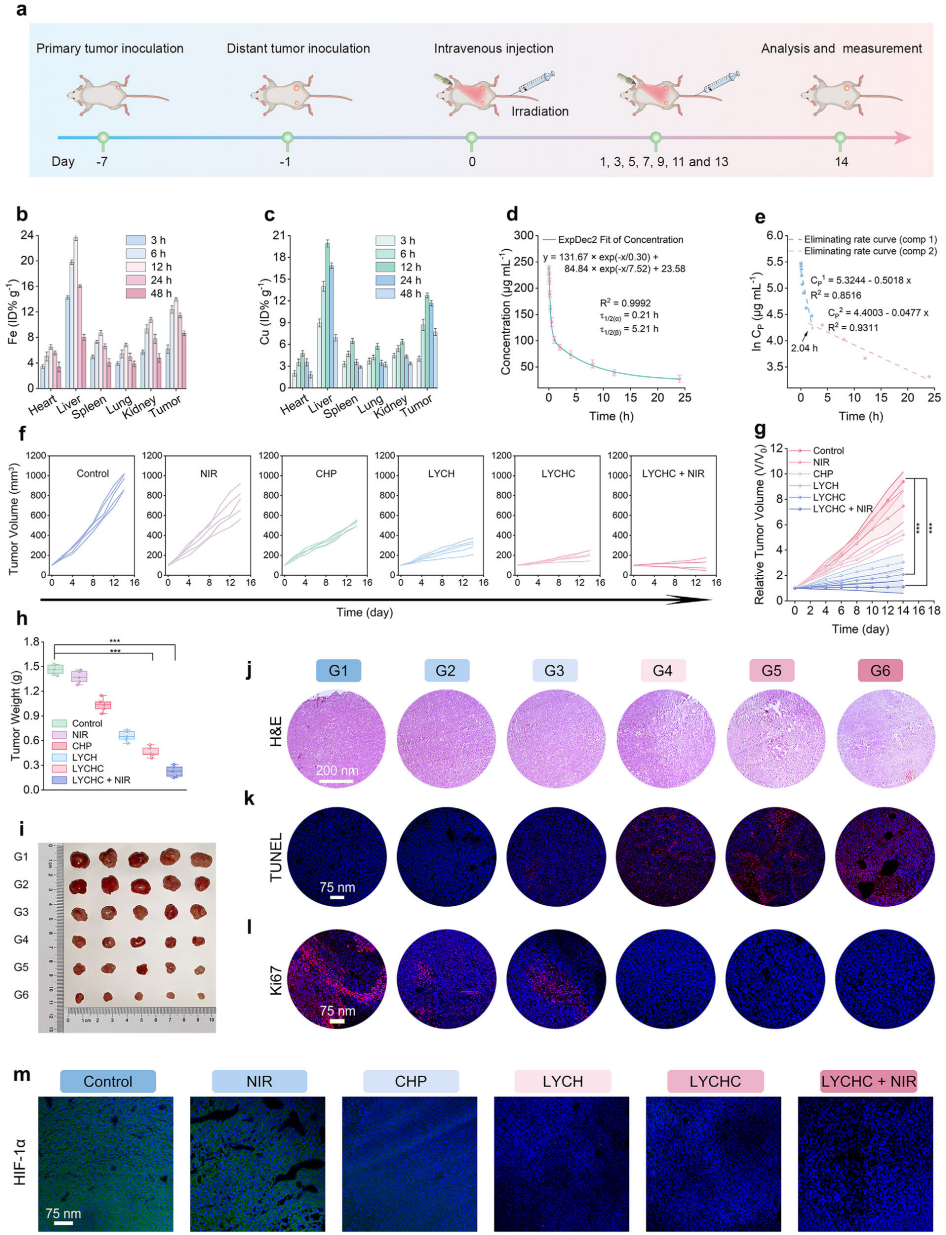
Primary tumor eradication via immunosuppressive microenvironment remodeling. (a) Schematic illustration of the treatment process. Biodistribution and accumulation of (b) Fe element and (c) Cu element in main organs and tumor after intravenous injections of LYCHC NPs (n = 3). (d) The blood circulation curve of the intravenously injected LYCHC NPs solution. (e) The elimination rate curve of the intravenously injected LYCHC NPs solution originated from (d). (f) Tumor volume growth, (g) relative tumor volume, (h) tumor weight, and (i) digital tumor photographs of the primary tumor after different treatments (n = 5). (j) H&E staining images of primary tumor tissue sections. Primary tumor tissue sections with (k) TUNEL staining, (l) Ki‐67 staining, and (m) HIF‐1α staining images. (G1: Control; G2: NIR; G3: CHP; G4: LYCH; G5: LYCHC; G6: LYCHC + NIR; ^*^p < 0.05, ^**^p < 0.01, ^***^p < 0.001, and *ns*: not significant).

Based on their excellent targeting and pharmacokinetic properties, the antitumor effects of LYCHC NPs were systematically evaluated. Dynamic monitoring of tumor volume during treatment revealed that the LYCHC + NIR group exhibited the strongest tumor growth inhibition (Figure 6f,g). Statistical analysis of tumor weights at the treatment endpoint further confirmed that the LYCHC + NIR group exhibited significantly lower tumor weights compared to other groups, while the NIR and control groups showed rapid tumor growth trends (Figure 6h). Digital photographs of primary tumors also visually demonstrated the differences in final treatment outcomes among groups (Figure 6i). Notably, even without NIR irradiation, the tumor suppression effect in the LYCHC NPs group remained significantly stronger than that in the CHP and LYCH groups. To further elucidate the in vivo antitumor mechanism of LYCHC NPs, tumor tissues were subjected to histological analysis and detection of specific molecular markers. Hematoxylin and eosin (H&E) staining images revealed varying degrees of nuclear condensation, dissolution, and tissue structural disruption in tumor cells across different treatment groups (Figure 6j). TUNEL staining results further confirmed that the LYCHC + NIR group exhibited significantly higher levels of apoptosis compared to other groups (Figure 6k). Concurrently, Ki‐67 immunofluorescence staining revealed the lowest positive cell rate in the LYCHC + NIR group, indicating effective suppression of tumor proliferative activity (Figure 6l). Crucially, immunofluorescence analysis of hypoxia‐inducible factor HIF‐1α revealed markedly downregulated HIF‐1α expression in the YC‐1‐containing LYCH group. The most pronounced suppression of HIF‐1α expression was observed in the LYCHC + NIR group (Figure 6m).

The favorable biocompatibility of LYCHC NPs has been validated through multidimensional safety assessments. In vivo continuous monitoring of mouse body weight during treatment revealed no significant weight loss in any treatment group compared with the control group, preliminarily ruling out severe systemic adverse effects induced by the treatment (Figure S21). H&E staining of major organs at the treatment endpoint showed no obvious histopathological alterations, such as inflammatory infiltration, cell necrosis, or fibrosis, in the LYCHC + NIR group, indicating that LYCHC NPs at the therapeutic dose do not cause irreversible structural damage to key metabolic and excretory organs (Figure S22). Furthermore, hemocompatibility assays demonstrated that the hemolysis rate of LYCHC NPs remained below the internationally recognized safety threshold of 5% at all tested concentrations, effectively avoiding the risks of acute kidney injury and immune disorders associated with intravascular hemolysis (Figure S23). Finally, as shown in Figure S24, all measured parameters, including white blood cell count, alanine aminotransferase, and creatinine, remained within normal ranges, indicating no overt hepatotoxicity or nephrotoxicity. Collectively, LYCHC NPs, while effectively activating robust antitumor immune responses, exhibit excellent in vitro and in vivo safety profiles by virtue of their ingenious responsive structure and metabolizable components, providing a reliable safety guarantee for the clinical translation of subsequent immunotherapy strategies based on the synergistic induction of ferroptosis and cuproptosis. By efficiently accumulating in tumor tissue via the EPR effect and acid‐responsive dissociation, LYCHC NPs under NIR irradiation synchronously activate multiple antitumor mechanisms, trigger intratumoral cascading cytotoxicity, and reverse the immunosuppressive microenvironment, which in turn establishes a favorable basis for systemic antitumor immunity.

### 2.7 Systemic Antitumor Immune Memory and Metastasis Suppression

The tumor immunotherapy strategy of LYCHC NPs not only requires eliminating the primary tumor but also aims to induce systemic, persistent antitumor immune memory to suppress tumor recurrence and distant metastasis. By promoting cytotoxic T lymphocyte infiltration and macrophage polarization toward the M1 phenotype, LYCHC NPs effectively establish long‐lasting immune surveillance, thereby inhibiting tumor recurrence and distant metastasis.

As shown in Figure S25 and Figure 7a, during treatment, the control and NIR groups exhibited sustained rapid growth in distant tumor volume, whereas the LYCHC + NIR group demonstrated the most flattened growth curve, indicating that LYCHC NPs significantly inhibit tumor progression. At the treatment endpoint, macroscopic tumor specimens and weight measurements further supported the conclusion (Figure 7b and Figure S26). As shown in Figure 7c, the LYCHC + NIR group achieved a high inhibition rate of approximately 61.15% for distant tumors, indicating that the therapeutic strategy not only effectively suppresses the growth of primary tumors but also induces systemic immune responses, thereby exerting significant inhibitory effects on distant metastatic lesions. In Figure 7d, LYCHC NPs induce ICD characterized by ferroptosis and cuproptosis, releasing large amounts of tumor antigens and DAMPs. Concurrently, LYCHC NPs alleviate tumor hypoxia by inhibiting the HIF‐1α signaling pathway, thereby enhancing antigen presentation capacity in DCs and T cell activation. Ultimately, they synergistically promote effector T cell activation, infiltration, and functional engagement at both local and systemic levels, reversing immunosuppression.

**FIGURE 7:**
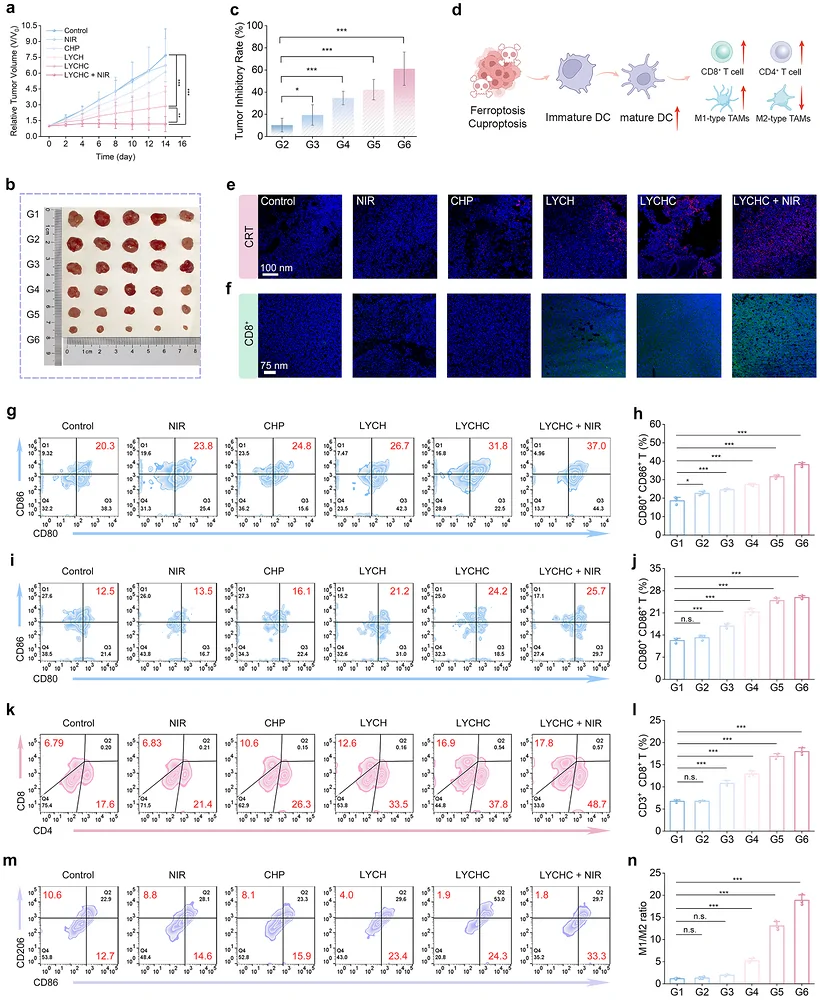
Systemic antitumor immune memory and metastasis suppression. (a) Relative tumor volume, (b) digital tumor photographs, and (c) tumor inhibitory rate of distant tumor after different treatments (n = 5). (d) Schematic diagram of reconstructing immunosuppressive TME. Primary tumor tissue sections (e) CRT staining and (f) CD8^+^ staining images. (g) Flow cytometry analysis and (h) semi‐quantitative analysis of CD80^+^ CD86^+^ cells in the draining lymph node. (i) Flow cytometry analysis and (j) semi‐quantitative analysis of CD80^+^ CD86^+^ cells in the spleens. (k) Flow cytometry analysis of CD4^+^ CD8^+^ cells in distant tumors in different groups. (l) The semiquantitative analysis of CD3^+^ CD8^+^ cells from (k). (m) Flow cytometry analysis of CD86^+^ CD206^+^ cells in the primary tumor in different groups. (n) The semiquantitative analysis of M1/M2 ratio from (m). (G1: Control; G2: NIR; G3: CHP; G4: LYCH; G5: LYCHC; G6: LYCHC + NIR; ^*^p < 0.05, ^**^p < 0.01, ^***^p < 0.001, and *ns*: not significant).

Immunological analysis of the primary tumor revealed significantly elevated levels of CRT exposure on the cell membrane in the tumor tissue of the LYCHC + NIR group, indicating widespread occurrence of ICD (Figure 7e). Concurrently, CD8^+^ T cell infiltration in the primary tumor tissue was markedly enhanced, suggesting that LYCHC NPs effectively promote the recruitment and activation of effector T cells to the tumor site (Figure 7f). A substantial release of DAMPs was promoted by LYCHC NPs, which significantly enhanced the immunogenicity of tumor cells and created favorable conditions for effective T cell recognition and killing. To further investigate the mechanism of systemic immune responses, the status of antigen‐presenting cells in draining lymph nodes and the spleen was evaluated. Flow cytometry analysis revealed a significant increase in the proportion of CD80^+^ CD86^+^ cells within draining lymph nodes in the LYCHC + NIR group, indicating that more antigen‐presenting cells were activated to a fully mature functional state (Figure 7g,h). Concurrently, the ratio of CD80^+^ CD86^+^ cells in the spleen also increased synchronously, further confirming that LYCHC NPs effectively activate systemic antitumor immune responses (Figure 7i,j). The successful reversal of the hypoxic TME by LYCHC NPs lifts its suppression on DCs function and maturation, thereby enhancing the efficiency of adaptive immune response initiation. Notably, in distant tumor tissues, both CD3^+^ CD8^+^ cytotoxic T cells and CD3^+^ CD4^+^ helper T cells showed significantly increased infiltration in the LYCHC + NIR group (Figure 7k,l and Figure S27). The nanomodulator LYCHC NPs not only activate immune responses at the primary tumor sites but also guide effector T cells to home toward distant tumor tissues, thereby establishing a complete immune circuit from antigen release and lymph node T cell activation to T cell recruitment at distant tumor sites. Phenotypic reprogramming of tumor‐associated macrophages plays a crucial role in remodeling the immunosuppressive microenvironment [62, 63]. Analysis of macrophage subpopulations in primary tumor tissues revealed that LYCHC + NIR treatment significantly increased the proportion of macrophages highly expressing CD86 (typically associated with the M1 phenotype) while decreasing the proportion of those highly expressing CD206 (typically associated with the M2 phenotype) (Figure 7m,n). The observed shift in macrophage polarization is likely attributable to LYCHC NPs‐mediated inhibition of the HIF‐1α pathway, by which they counteract the M2‐polarizing effect of tumor hypoxia. Furthermore, LPO and other oxidative stress signaling molecules generated during ferroptosis and cuproptosis may synergistically promote the phenotypic conversion of macrophages toward an anti‐tumor functional state [64, 65].

Overall, mice treated with LYCHC NPs exhibited significant suppression of distant tumor growth, demonstrating that the LYCHC + NIR therapeutic strategy can induce effective systemic antitumor immunity. Comprehensive alterations in immunological markers clearly reveal that LYCHC NPs successfully transform “cold” tumors into “hot” tumors by remodeling the immunosuppressive microenvironment, thereby establishing robust antitumor immune memory.

## 3 Conclusion

In summary, by cascadingly triggering ferroptosis and cuproptosis pathways, LYCHC NPs achieve the activation of ICD and the systemic remodeling of the immunosuppressive microenvironment. Using TME‐specific response mechanisms, LYCHC NPs enable spatiotemporal coordination of multiple therapeutic modes under NIR laser regulation, thereby overcoming limitations of conventional immunotherapy. Following tumor cell internalization, LYCHC NPs first induce mitochondrial calcium overload via acid‐triggered Ca^2+^ release, leading to mitochondrial membrane potential collapse and cellular energy metabolism disruption. Concurrently, the photothermal effect not only directly kills tumor cells but also serves as a key trigger signal, accelerating NO production and iron/copper ion release. The large amounts of ROS and RNS significantly exacerbate LPO and deplete intracellular GSH reserves. Notably, released copper ions specifically target acylated mitochondrial enzymes, disrupting normal tricarboxylic acid cycle function. Ultimately, LYCHC NPs simultaneously activate both ferroptosis and cuproptosis death pathways. The induction of a dual immunogenic death pattern prompts damaged tumor cells to release multiple DAMPs, including HMGB1, ATP, and CRT, effectively promoting DCs' maturation and antigen presentation. Experiments confirm that the LYCHC + NIR treatment strategy significantly enhances cytotoxic T lymphocyte infiltration in tumor tissues and induces macrophage polarization toward the M1 phenotype, thereby successfully transforming immunosuppressive “cold” tumors into immunologically active “hot” tumors. Additionally, by employing NO gas therapy and the YC‐1 drug to inhibit the HIF‐1α signaling pathway, the nanomodulator effectively alleviates the hypoxic tumor microenvironment, further enhancing the activity and function of immune cells. In animal models, LYCHC + NIR therapy not only achieves effective clearance of primary tumors but also establishes durable systemic antitumor immune memory, significantly suppresses tumor recurrence and distant metastasis. In summary, the avalanche effect therapy strategy based on self‐amplification achieves fundamental remodeling of the immunosuppressive microenvironment by integrating multiple therapeutic modules, including calcium overload, gas therapy, photothermal synergy, and dual‐metal‐induced cell death.

## 4 Experimental Section

### 4.1 Synthesis of CHP NPs

For the synthesis of PB NPs, 12.0 g of Poly(vinylpyrrolidone) (PVP, K30) and 528.0 mg of K_3_[Fe(CN)_6_]·3H_2_O were dissolved in 160 mL of HCl (0.01 M). The mixture was maintained at 20 °C, 40 °C, and 60 °C for 2 h at each temperature, followed by heating in an oven at 80 °C for 15 h. The resulting blue product, PB NPs, was collected by centrifugation (12,000 rpm, 10 min) and washed three times alternately with anhydrous ethanol and deionized water. Subsequently, following a reported procedure, 2.0 mg of PB NPs and 10.0 mg of PVP were dissolved in 10 mL of HCl (1 M). The mixture was stirred at room temperature for 2 h and then heated at 140 °C for 3 h. The resulting product, HPB NPs, was isolated by centrifugation (12,000 rpm, 10 min). Next, 22.0 mg of Cu(CH_3_COO)_2_·H_2_O, 67.5 mg of trisodium citrate dihydrate, and 250.0 mg of PVP were added to 20 mL of deionized water. The solution was mixed with 50.0 mg of HPB NPs and stirred at room temperature for 3 h. Then, 66.0 mg of K_3_[Fe(CN)_6_]·3H_2_O was added, and stirring was continued for 24 h to obtain CHP NPs.

### 4.2 Synthesis of LYCHC NPs

Initially, 50.0 mg of dried CHP NPs powder and 25.0 mM of L‐Arg were dissolved in 50 mL of deionized water, and the mixture was stirred at room temperature for 2 h. Subsequently, 10.0 mM of YC‐1 was introduced, and stirring continued for 16 h. The resulting product, denoted as LYCH NPs, was collected via centrifugation (8,000 rpm, 10 min) and dried for 24 h. For comparison, control samples (CHP + L‐Arg and CHP + YC‐1) were prepared by adding only one of the two agents (either L‐Arg or YC‐1) to the CHP dispersion under otherwise identical stirring conditions, i.e., stirring for 2 h without the subsequent addition of the other component. To obtain LYCHC NPs, 14.8 mg of Ca(OH)_2_ and 32.0 mg of poly (acrylic acid) were added to 40 mL of deionized water containing the as‐synthesized LYCH NPs, and the mixture was stirred at room temperature for 1 h. A solution of 76 mL of isopropanol and 22.5 mg of (NH_4_)_2_HPO_4_ was rapidly introduced into the mixture. Stirring was maintained for an additional 24 h. The final product, LYCHC NPs, was isolated by centrifugation (8,000 rpm, 10 min) and subjected to a 24 h drying process.

### 4.3 Evaluation of the Photothermal Effect of LYCHC NPs

The photothermal performance of LYCHC NPs was evaluated by monitoring the temperature change using an infrared thermal imaging camera. Specifically, the temperature rise was recorded under irradiation with varying laser power densities (0.6, 0.8, 1.0, and 1.2 W cm^−2^) at a fixed LYCHC NPs concentration, and also at different concentrations (25, 50, 100, and 200 µg mL^−1^) under a fixed laser power. Furthermore, the photothermal stability was assessed over four laser on/off cycles, and the photothermal conversion efficiency (*η*) was calculated.

### 4.4 Cell Culture

L929 fibroblast cells (RRID: CVCL_0462) and CT26 mouse colon carcinoma cells (RRID: CVCL_7256) were procured from Zhejiang Mason Cell Technology and Beyotime Biotechnology, respectively. All cell lines were verified to be free of mycoplasma contamination. Cells were cultured in Dulbecco's modified Eagle's medium (DMEM) supplemented with 10% fetal bovine serum (FBS) and 1% penicillin‐streptomycin, and maintained at 37 °C in a humidified atmosphere containing 5% CO_2_.

### 4.5 Detection of Intracellular HIF‐1α, DLAT, CRT, and HMGB1 Protein Expression

CT26 cells were cultured in 6‐well plates for 12 h. The cells were then treated with different materials (100 µg mL^−1^) for 4 h. After treatment, the cells were fixed with 4% polyformaldehyde for 20 min and subjected to immunostaining. Specifically, cells were incubated overnight at 4 °C with anti‐HIF‐1α antibody, anti‐DLAT antibody, anti‐CRT antibody, and anti‐HMGB1 antibody. Following PBS washes, the cells were incubated with fluorophore‐conjugated secondary antibodies for 1 h at room temperature.

### 4.6 Animal Tumor Models

BALB/c mice were obtained from Guangxi Medical University Animal Experiment Center (China). All animal procedures were performed in accordance with the Guide for the Care and Use of Laboratory Animals and the guidelines of the Institutional Animal Care and Ethics Committee of Guangxi Medical University Cancer Hospital (protocol code KY2023177; approved on January 1, 2024). To establish the tumor model, CT26 cells (2 × 10^6^) suspended in 100 µL of PBS were subcutaneously injected into the right flank of each mouse. Six days later, the same number of cells was inoculated into the left flank. The mice were randomly divided into six groups (n = 5 per group): G1 (Control), G2 (NIR), G3 (CHP), G4 (LYCH), G5 (LYCHC), and G6 (LYCHC + NIR). Treatments were administered via tail vein injection on days 1, 3, 5, 7, 9, 11, and 13. Following injection, mice in the G6 group were exposed to 808 nm laser irradiation (1.0 W cm^−2^) for 10 min. Tumor dimensions and body weight were measured every two days throughout the treatment period.

### 4.7 Histological Analysis and Immunofluorescence Staining

At the endpoint of the treatment, tumor tissues from each group were collected, fixed in 4% polyformaldehyde, embedded in paraffin, and sectioned into 4 µm slices. The sections were deparaffinized, rehydrated, and subjected to antigen retrieval in citrate buffer. The staining procedures were divided into two parallel pathways: one set of sections was stained with H&E and processed for TUNEL assay according to standard protocols for histopathological evaluation. The other set was used for immunofluorescence staining, which involved sequential incubation with antibodies (Ki‐67, HIF‐1α antibody, CRT, HMGB1, and CD8^+^ antibody) at 4 °C overnight, followed by corresponding fluorophore‐conjugated secondary antibodies for 2 h at room temperature. Nuclei were counterstained with DAPI. All sections were washed with PBS and imaged under CLSM.

### 4.8 Flow Cytometric Analysis of Immune Cells In Vivo

After 14 days of treatment, mice from each group were euthanized, and the spleen, lymph nodes, and tumor tissues were collected. Single‐cell suspensions were prepared by mechanical dissociation and filtration through a 70 µm strainer. After washing with PBS, immune cells were isolated using density gradient centrifugation and subsequently stained with fluorescently labeled antibodies for surface and intracellular markers. Cell populations were analyzed by flow cytometry (Attune NxT), and data were processed with FlowJo software.

### 4.9 Statistical Analysis

Data are presented as mean ± SD from at least three independent experiments. Multiple comparisons between groups were performed using one‐way analysis of variance and Bonferroni's post‐hoc test in GraphPad Prism 9.5 software. A *p*‐value of less than 0.05 was considered statistically significant, with specific thresholds denoted as ^*^p < 0.05, ^**^p < 0.01, ^***^p < 0.001, and ns (not significant).

## Author Contributions

**Hongmei Zhou**: data curation, formal analysis, visualization, Writing – original draft, Writing – review and editing, investigation, methodology. **Guilan Xu**: data curation, formal analysis, visualization, methodology, investigation, validation, writing – original draft. **Yulin Liang**: data curation, formal analysis, investigation, validation, methodology, visualization, writing – original draft. **Caixi Yu**: data curation, formal analysis, investigation, methodology, validation, visualization, writing – original draft. **Yujia Liang**: data curation, investigation, methodology, writing – original draft, funding acquisition. **He Ding**: conceptualization, formal analysis, investigation, project administration, supervision, funding acquisition, writing – review and editing, resources. **Lijuan Liu**: data curation, formal analysis, validation, writing – original draft. **Litu Zhang**: data curation, formal analysis, investigation, methodology, project administration, supervision, validation, visualization, writing – review and editing. **Piaoping Yang**: writing – review and editing, supervision, project administration, conceptualization, investigation, resources. **Chen Wang**: conceptualization, investigation, supervision, funding acquisition, project administration, writing – review and editing, resources.

## Conflicts of Interest

The authors declare no conflict of interest.

## Supporting information




**Supporting File**: advs77120‐sup‐0001‐SuppMat.docx.

## Data Availability

The data that support the findings of this study are available from the corresponding author upon reasonable request.
