# Supplementary material for: An Avalanche Effect Nanomodulator Triggers Cascade Ferroptosis/Cuproptosis for Immunosuppressive Microenvironment Remodeling
Source: Adv Sci (Weinh). 2026 Aug 13;13(61):e77120. doi: 10.1002/advs.77120 (PMC13472115; doi:10.1002/advs.77120)
Supplement: Supplementary file 1 [file ADVS-13-e77120-s001.docx]

Supporting Information

**An Avalanche Effect Nanomodulator Triggers Cascade Ferroptosis/Cuproptosis for Immunosuppressive Microenvironment Remodeling**

*Hongmei Zhou, Caixi Yu, Guilan Xu, Lijuan Liu, Yujia Liang, Yulin Liang, He Ding*, Litu Zhang*, Piaoping Yang*, and Chen Wang**

**Experimental Section**

*Materials and Reagents:* Poly(vinylpyrrolidone) (PVP, K30), K_3_[Fe(CN)_6_]·3H_2_O, Cu(CH_3_COO)_2_·H_2_O, trisodium citrate dihydrate, L-arginine (L-Arg), poly(acrylic acid), Cy5-PEG-NH_2_, 3,3',5,5'-tetramethylbenzidine (TMB), nitroblue tetrazolium chloride (NBT), 5,5-dimethyl-1-pyrroline N-oxide (DMPO), glutathione (GSH), 5,5'-dithiobis-2-nitrobenzoic acid (DTNB), 2,2,6,6-tetramethylpiperidine (TEMP), and 1,3-diphenylisobenzofuran (DPBF) were purchased from Aladdin Reagent Co., Ltd. (Shanghai, China). Lificiguat (YC-1) was obtained from McLean Biochemicals Co., Ltd. (Shanghai, China). Calcium hydroxide (Ca(OH)_2_), 9,10-anthracenediyl-bis(methylene)dimalonic acid (ABDA), and diammonium hydrogen phosphate [(NH_4_)_2_HPO_4_] were acquired from Sinopharm Chemical Reagent Co., Ltd. (Shanghai, China). Penicillin-streptomycin solution, fetal bovine serum (FBS), phosphate-buffered saline (PBS), ferrostatin-1 (Fer-1), Dulbecco's modified Eagle's medium (DMEM), the Griess reagent kit, the MTT cell proliferation and cytotoxicity assay kit, 2',7'-dichlorodihydrofluorescein diacetate (DCFH-DA), singlet oxygen sensor green (SOSG), dihydroethidium (DHE), tris(4,7-diphenyl-1,10-phenanthroline)ruthenium(II) dichloride ([Ru(dpp)_3_]Cl_2_), Fluo-4 acetoxymethyl ester (Fluo-4 AM), 4-amino-5-methylamino-2',7'-dichlorofluorescein diacetate (DAF-FM DA), acridine orange (AO), KT5823, GSH assay kits, malondialdehyde (MDA) assay kit, Hoechst 33342 staining solution, calcein acetoxymethyl ester (Calcein AM)/propidium iodide (PI) cell viability/cytotoxicity assay kit, mitochondrial membrane potential assay kit with JC-1, Annexin V-FITC/PI apoptosis assay kit, hematoxylin and eosin (H&E) staining kit, Ki67 cell proliferation detection kit (immunofluorescence, red, rabbit monoclonal antibody), and one step terminal deoxynucleotidyl transferase-mediated dUTP-digoxigenin nick-end labeling (TUNEL) apoptosis detection kit were acquired from Beyotime Biotechnology Co., Ltd. (Shanghai, China). Fluorescein isothiocyanate (FITC) was obtained from Shanghai Yien Chemical Technology Co., Ltd. (Shanghai, China). The anti-GPX4 antibody, liperfluo, CD11c monoclonal antibody, CD80 (B7-1) monoclonal antibody, CD86 (B7-2) monoclonal antibody, CD45 monoclonal antibody, CD3e monoclonal antibody, CD4 monoclonal antibody, CD8a monoclonal antibody, F4/80 monoclonal antibody, and CD206 (MMR) monoclonal antibody were purchased from Thermo Fisher Scientific (USA). The ATP assay kit, phenylmethanesulfonyl fluoride, phosphatase inhibitor cocktail, high-efficiency RIPA lysis buffer, and TBST buffer were purchased from Solarbio Science and Technology Co., Ltd. (Beijing, China). The peroxynitrite (ONOO^−^) probe was acquired from Shanghai Pebble Biotechnology Co., Ltd. (Shanghai, China). Anti-DLAT primary antibody, Anti-GPX4 primary antibody, Anti-HIF-1α primary antibody, Anti-HMGB1 primary antibody, and Anti-CRT primary antibody were obtained from Proteintech Co., Ltd. (Wuhan, China). One-step PAGE gel rapid preparation kit, and a prestained protein marker ranging from 10 to 250 kDa were purchased from EpiZyme Co., Ltd. (Shanghai, China). The other reagents in this work were used without further purification.

*Characterization*: X-ray diffraction was performed on a Bruker D8 Advance diffractometer (Germany). Transmission electron microscopy (TEM) was conducted using an FEI TALOS G2 F200X microscope (Czech Republic). The surface zeta potential and hydrodynamic size of the nanoparticles were determined by dynamic light scattering using a Zetasizer Nano ZS analyzer (UK). Nitrogen adsorption-desorption isotherms were measured at 77 K using a Micromeritics 3FLEX surface characterization analyzer (USA) to calculate the specific surface area and pore size distribution via the Brunauer-Emmett-Teller and Barrett-Joyner-Halenda methods, respectively. X-ray photoelectron spectroscopy analysis was carried out on a Thermo Scientific Escalab Xi+ spectrometer (USA). Fourier transform infrared spectroscopy was performed on a Thermo Scientific Nicolet iS20 spectrometer to identify surface functional groups. UV−Vis spectroscopy was conducted using a Shimadzu UV-2600 spectrophotometer (Japan). The release kinetics of Cu and Fe ions were quantified by an iCAP 7400 inductively coupled plasma optical emission spectrometer (ICP-OES). An inductively coupled plasma-mass spectrometer (ICP-MS, Thermo Fisher, USA) was used to determine the content of the specified ions in animal organs. The photothermal performance was monitored with an NEC Avio R300SR-HD infrared thermal imaging camera (Japan). Confocal laser scanning microscopy (CLSM) images were acquired on a Leica SP8 microscope (Germany). Apoptosis analysis was performed using a BD Accuri C6 flow cytometer (USA). Flow Cytometric Analysis of Immune Cells was performed using Attune NxT (Thermo Fisher Scientific). Western blot analysis was performed using a Jena UVP ChemStudio815 imaging system. Fluorescence imaging of mice in vivo was conducted on a PerkinElmer Lumina XRMS Series Ⅲ imaging system.

*Drug Release Behavior of L-Arg and YC-1*: To investigate the release of nitric oxide (NO) from L-Arg, LYCHC NPs were dispersed in PBS solutions with varying pH levels (5.0, 6.5, and 7.4) containing 1 mM H_2_O_2_. The NO concentration in the supernatant was quantified over time using a Griess reagent kit, according to the manufacturer's protocol. Absorbance was measured at 540 nm, and the concentration was determined using a standard curve. Simultaneously, the release of YC-1 was monitored. A standard curve for free YC-1 was established in DMSO by measuring the absorbance at 328 nm. The amount of YC-1 released from LYCHC NPs under different conditions was then calculated based on this standard curve.

*Assessment of ROS (·OH, ^1^O_2_, and ·O_2_^−^) Generation*: The capacity of LYCHC NPs to generate ·OH was investigated using the chemical probe TMB. The PBS reaction mixture containing TMB (2 mM) and H_2_O_2_ (10 mM) was incubated with the test sample (100 μL, 1 mg mL^−1^) for 5 min, and the absorbance at 652 nm was monitored under different temperature conditions using UV–vis spectroscopy. Additionally, the absorbance of the reaction solution at 652 nm was recorded at various time intervals by varying the reaction time. To quantify the catalytic efficiency of LYCHC NPs toward H_2_O_2_, the nanoparticles (100 μL, 1 mg mL^−1^) were reacted with varying concentrations of H_2_O_2_ (2.5, 5, 10, 20, and 40 mM). The change in absorbance at 652 nm was recorded over a 5-min period. The resulting kinetic data were analyzed using Michaelis-Menten models and Lineweaver-Burk plots. The production of ·O_2_^−^ was assessed via NBT, which forms an insoluble blue formazan precipitate upon reaction with ·O_2_⁻, exhibiting a characteristic absorption peak at 560 nm. NBT (779.8 μM) was introduced into a solution containing LYCHC NPs (100 μL, 1 mg mL^−1^) and H_2_O_2_ (10 mM), and the time-dependent increase in absorbance at 560 nm was measured at intervals (0, 2, 4, 6, 8, and 10 min). For the detection of ^1^O_2_, ABDA was employed as a probe. A mixture of LYCHC NPs (100 μL, 1 mg mL^−1^) and ABDA solution (100 μL, 4 mg mL^−1^) in 3 mL of PBS was prepared, and the decay of the ABDA absorbance was monitored by UV−Vis spectroscopy at specified time points (0, 2, 4, 6, 8, and 10 min). ESR spectroscopy was used to corroborate the generation of ROS. DMPO served as the spin trapping agent for ·OH and ·O_2_^−^, while TEMP was used for ^1^O_2_. A solution containing 10 μL of LYCHC NPs (100 μg mL^−1^) was added to a mixture of 10 μL of the DMPO/TEMP and 10 μL of H_2_O_2_ (10 mM). The final solution was drawn into a quartz capillary, and ESR spectra were acquired at both room temperature and 45 °C to identify the specific ROS.

*Assessment of GSH Depletion*: The depletion of GSH was evaluated by incubating LYCHC NPs (100 μg mL^−1^) with GSH (1.0 mM) in PBS solutions at different pH values (5.0, 6.5, and 7.4). Subsequently, DTNB was introduced into the mixture at a final concentration of 0.05 mM to quantify the remaining GSH levels.

*Quantification of O_2_ Generation*: O_2_ generation was determined by measuring the concentration of dissolved O_2_ using a portable dissolved O_2_ meter (JPBJ-608, Leici). LYCHC NPs (100 μg) were added to 2 mL of 200 mM H_2_O_2_, and the O_2_ concentration was monitored over 1 min under various conditions. A control experiment using pure H_2_O_2_ (200 mM) was conducted in parallel to account for background O_2_ generation from the spontaneous decomposition of H_2_O_2_.

*Cellular Uptake Assay*: CT26 cells were seeded into 6-well plates at a density of 1 × 10^5^ cells per well. For the uptake experiment, cells were incubated with FITC-labeled LYCHC NPs (100 µg mL^−1^) for varying time intervals (1, 2, 3, and 4 h). Following incubation, the cells were washed three times with PBS and stained with Hoechst 33342 before imaging by CLSM.

*In Vitro Cytotoxicity Assessment*: The cytotoxicity of LYCHC NPs against CT26 cells and their biocompatibility with L929 cells were evaluated using the MTT assay. Briefly, CT26 cells were treated according to different experimental groups (Control, NIR, CHP, LYCH, LYCHC, and LYCHC + NIR) and exposed to varying concentrations of nanoparticles (0, 25, 50, 100, 200, and 400 μg mL^−1^) for 24 h. In the LYCHC + NIR group, cells were irradiated with an 808 nm laser (1.0 W cm^−2^, 10 min) following incubation. After treatment, the medium was replaced, and 20 μL of MTT solution (5 mg mL^−1^) was added to each well, followed by further incubation for 4 h. Similar steps were applied to L929 cells to assess the biosafety of the nanoparticles.

*Detection of Intracellular ROS*: CT26 cells seeded in 6-well plates were divided into six experimental groups: G1 (Control), G2 (NIR), G3 (CHP), G4 (LYCH), G5 (LYCHC), and G6 (LYCHC + NIR). After 4 h of treatment under respective conditions, cells were stained with DCFH-DA, SOSG, or DHE probes for 20 min to detect total ·OH, ^1^O_2_, and ·O_2_^−^, respectively. Fluorescence images were acquired using CLSM. Additionally, CT26 cells stained with DCFH-DA were harvested for quantitative analysis by flow cytometry.

*Evaluation of Intracellular O_2_ Levels*: The O_2_ generation capability of LYCHC NPs upon 808 nm laser irradiation was examined in vitro using the [Ru(dpp)_3_]Cl_2_ fluorescent probe. Cells were pre-treated with LYCHC NPs for 4 h, followed by staining with [Ru(dpp)_3_]Cl_2_. Fluorescence images were subsequently captured by CLSM to visualize intracellular O_2_ levels.

*Detection of Intracellular Ca^2+^ Levels*: CT26 cells were seeded in 6-well plates and cultured overnight. After washing with PBS, the cells were treated with LYCHC NPs (100 µg mL^−1^) for 1, 2, 3, and 4 h. During the treatment period, the LYCHC + NIR group was subjected to concurrent irradiation with an 808 nm laser (1.0 W cm^−2^, 10 min). To visualize intracellular Ca^2+^, cells were stained with Fluo-4 AM for 20 min, rinsed with PBS, and subsequently co-stained with Hoechst 33342 for 10 min before imaging by CLSM.

*Detection of Intracellular RNS*: Intracellular RNS was detected using the DAF-FM DA probe (for NO) and an ONOO^−^ probe. Briefly, CT26 cells were seeded in 6-well plates and cultured for 12 h. The culture medium was then replaced with fresh medium containing different materials (100 µg mL^−1^), followed by another 4 h of incubation. The LYCHC + NIR group was irradiated with an 808 nm laser during this period. After washing with PBS, the cells were incubated with 1 mL of DAF-FM DA or ONOO^−^ probe staining solution for 20 min. Unincorporated dye was removed by PBS washing before CLSM imaging.

*Assessment of Mitochondrial Membrane Potential*: CT26 cells were treated following the same protocol as described for the intracellular RNS assay. Subsequently, the cells were incubated with JC-1 staining solution according to the manufacturer's instructions and visualized under CLSM. Mitochondrial membrane potential was evaluated based on the fluorescence shift from red J-aggregates (indicating high potential) to green J-monomers (indicating low potential).

*Assessment of Intracellular LPO*: CT26 cells were seeded in 6-well plates. Following the different treatments, cells were stained with liperfluo dye (a sensitive fluorescent probe for LPO for 30 min and subsequently imaged by CLSM.

*Measurement of Intracellular MDA, GSH, and ATP Levels*: CT26 cells were plated in 96-well plates and cultured overnight. After a 4 h incubation under different treatment conditions, cells were harvested. The intracellular levels of MDA, GSH, and ATP were then quantified using their respective commercial assay kits according to the manufacturers' protocols.

*Assessment of Cell Apoptosis*: CT26 cells were seeded in 6-well and 96-well plates and divided into six experimental groups: G1 (Control), G2 (NIR), G3 (CHP), G4 (LYCH), G5 (LYCHC), and G6 (LYCHC + NIR). Cells were treated with the respective materials (100 μg mL^−1^) for 4 h. The G6 group was irradiated with an 808 nm laser (1.0 W cm^−2^, 10 min) following incubation. For live/dead staining, cells were sequentially treated with PBS, AM (calcein-AM), PI (propidium iodide), PBS, 2.5% glutaraldehyde, and PBS in the dark, and then visualized by CLSM. For quantitative apoptosis analysis, cells were processed according to the manufacturer's protocol provided with the Annexin V-FITC/PI apoptosis detection kit. Apoptotic populations were analyzed using a BD Accuri C6 flow cytometer.

*Western Blotting Analysis of GPX4 and DLAT Expression*: CT26 cells were seeded in 6 well plates and treated with different formulations, including Control, CHP, CHP + L‑Arg, CHP + YC‑1, LYCH, and LYCHC + NIR at 100 μg mL^−1^ for 4 h. After treatment, cells were lysed in RIPA buffer containing protease and phosphatase inhibitors. Protein concentrations were determined using a BCA assay. Equal amounts of protein were separated by SDS polyacrylamide gel electrophoresis and transferred onto polyvinylidene fluoride membranes. The membranes were blocked with 5% non fat milk for 1 h at room temperature and then incubated overnight at 4°C with primary antibodies against GPX4 and DLAT. After washing with TBST, the membranes were incubated with horseradish peroxidase-conjugated secondary antibodies for 1 h at room temperature. Protein bands were visualized using enhanced chemiluminescence ECL substrate and imaged with the Jena UVP ChemStudio815 imaging system.

*Western Blotting Analysis of HIF‑1α Expression with cGMP‑PKG Pathway Inhibition*: CT26 cells were pretreated with 150 μM CoCl_2_ for 4 h to establish hypoxic conditions, then divided into Control, LYCHC + NIR, and LYCHC + NIR + KT5823 groups. Cells in the LYCHC + NIR groups were treated with 100 μg mL^−1^ LYCHC NPs followed by 808 nm laser irradiation at 1.0 W cm^−2^ for 10 min. After 4 h of incubation, cells were lysed in RIPA buffer with protease and phosphatase inhibitors. Protein concentration was determined by the BCA assay. Equal amounts of protein were separated by SDS-PAGE and transferred to PVDF membranes. After blocking with 5% non fat milk, membranes were incubated with anti‑HIF‑1α primary antibody overnight at 4°C, followed by HRP-conjugated secondary antibody for 1 h at room temperature. Protein bands were visualized using an enhanced chemiluminescence substrate.

*In Vivo Fluorescence Imaging for Biodistribution*: CT26 tumor-bearing mice were intravenously injected with Cy5-labeled LYCHC NPs at a dose of 10 mg kg^−1^ via the tail vein. Fluorescence images were acquired using an in vivo imaging system at 2, 4, 8, 12, 24, and 48 h post-injection.

*Biodistribution Study*: CT26 tumor-bearing mice were intravenously administered LYCHC NPs (10 mg kg^−1^) and sacrificed at 3, 6, 12, 24, and 48 h post-injection. Major organs (heart, liver, spleen, lungs, and kidneys) and tumors were harvested, weighed, and digested in concentrated nitric acid. The concentrations of iron and copper in each tissue were quantified using ICP-MS.

*Pharmacokinetic Analysis*: CT26 tumor-bearing mice (n = 3) were intravenously injected with LYCHC NPs (10 mg kg^−1^). Blood samples were collected at 0, 10, 20, 30, 40, and 50 min, as well as at 1, 2, 3, 6, 12, and 24 h post-administration. The concentration of LYCHC NPs in circulation was determined by measuring the relevant metal content via ICP-MS.

**Figure S1.** (a) N_2_ adsorption-desorption isotherms of CHP and LYCH NPs. Pore diameter distribution of (b) CHP and (c) LYCH NPs.

**Figure S2.** TEM image of LYCHC NPs.

**Figure S3.** Energy dispersive spectroscopy survey spectrum of LYCHC NPs.

**Figure S4.** Particle size distribution diagram of LYCHC NPs.

**Figure S5.** PDI of LYCHC NPs dispersed in different solutions and hydrodynamic diameter (DLS) of LYCHC NPs in PBS over time. Data were displayed as mean ± S.D (n = 3).

**Figure S6.** Standard curve for YC-1 concentration.

**Figure S7.** Standard curve for NO concentration.

**Figure S8.** UV−Vis spectra of LYCHC NPs at different concentrations.

**Figure S9.** (a) Temperature variations and (b) infrared thermal spectra of LYCHC NPs under different NIR irradiation powers.

**Figure S10.** Effect of different concentrations on the photothermal performance of LYCHC NPs.

**Figure S11.** Single heating/cooling cycle of LYCHC NPs solution under NIR irradiation (1.0W cm^−2^, 100 μg mL^−1^).

**Figure S12.** Concentration-dependent absorbance spectra of oxidized TMB in the presence of H_2_O_2_ and LYCHC NPs.

**Figure S13.** Michaelis–Menten curves of LYCHC NPs at 25 °C and 45 °C, respectively.

**Figure S14.** JC-1 staining of CT26 cells with different treatments.

**Figure S15.** Cell viability of CT26 cells in different groups (**p* < 0.05, ***p* < 0.01, ****p* < 0.001, and *ns*: not significant).

**Figure S16.** CLSM images stained with HIF-1α probe after different treatments.

**Figure S17.** Immunofluorescence images of CT26 cells stained with anti-DLAT antibody.

**Figure S18.** Western blotting for GPX4 and DLAT protein expression in CT26 cells. (G1: Control; G2: CHP; G3: CHP + L-Arg; G4: CHP + YC-1; G5: LYCH; G6: LYCHC + NIR).

**Figure S19.** Western blotting for HIF-1α protein expression in CT26 cells. (G1: Control; G2: LYCHC + NIR; G3: LYCHC + NIR + KT5823).

**Figure S20.** In vivo fluorescence images of CT26 tumor-bearing mice at different time points after tail vein injection of Cy5-labeled LYCHC NPs.

**Figure S21.** Body weights of mice in different groups during the treatment period.

**Figure S22.** H&E staining of normal tissues (heart, liver, spleen, lung, and kidney) in different groups.

**Figure S23.** Hemolysis analysis of blood incubated with water (positive control) and various concentrations of LYCHC NPs (inset shows the corresponding digital photograph).

**Figure S24.** Hematological and blood biochemistry analysis of mice in LYCHC + NIR group (n = 3).

**Figure S25.** Tumor volume growth of distant tumor after different treatments (G1: Control; G2: NIR; G3: CHP; G4: LYCH; G5: LYCHC; G6: LYCHC + NIR).

**Figure S26.** Tumor weight of distant tumor after different treatments (**p* < 0.05, ***p* < 0.01, ****p* < 0.001, and *ns*: not significant).

**Figure S27.** The semiquantitative analysis of CD3^+^ CD4^+^ T cells in distant tumors after in vivo treatment (G1: Control; G2: NIR; G3: CHP; G4: LYCH; G5: LYCHC; G6: LYCHC + NIR; **p* < 0.05, ***p* < 0.01, ****p* < 0.001, and *ns*: not significant).
